# Genomic-based phylogenetic and metabolic analyses of the genus *Natronomonas*, and description of *Natronomonas aquatica* sp. nov.

**DOI:** 10.3389/fmicb.2023.1109549

**Published:** 2023-01-20

**Authors:** Alicia García-Roldán, Ana Durán-Viseras, Rafael R. de la Haba, Paulina Corral, Cristina Sánchez-Porro, Antonio Ventosa

**Affiliations:** ^1^Department of Microbiology and Parasitology, Faculty of Pharmacy, University of Sevilla, Sevilla, Spain; ^2^Department of Biology, University of Naples Federico II, Naples, Italy

**Keywords:** *Natronomonas*, hypersaline environments, salterns, extremophiles, haloarchaea, metabolism, phylogenomics, polar lipids

## Abstract

The genus *Natronomonas* is classified on the family *Haloarculaceae*, within the class *Halobacteria* and currently includes six species isolated from salterns, saline or soda lakes, and salt mines. All are extremely halophilic (optimal growth at 20–25% [w/v] NaCl) and neutrophilic, except *Natronomonas pharaonis*, the type species of the genus, that is haloalkaliphilic (showing optimal growth at pH 9.0) and possesses distinct phenotypic features, such as a different polar lipid profile than the rest of species of the genus. We have carried out a genome-based study in order to determine the phylogenetic structure of the genus *Natronomonas* and elucidate its current taxonomic status. Overall genomic relatedness indexes, i.e., OrthoANI (Average Nucleotide Identity), dDDH (digital DNA–DNA hybridization), and AAI (Average Amino acid Identity), were determined with respect to the species of *Natronomonas* and other representative taxa of the class *Halobacteria.* Our data show that the six species of *Natronomonas* constitute a coherent cluster at the genus level. Besides, we have characterized a new haloarchaeon, strain F2-12^T^, isolated from the brine of a pond of a saltern in Isla Cristina, Huelva, Spain, and we determined that it constitutes a new species of *Natronomonas*, for which we propose the name *Natronomonas aquatica* sp. nov. Besides, the metabolic analysis revealed a heterotrophic lifestyle and a versatile nitrogen metabolism for members of this genus. Finally, metagenomic fragment recruitments from a subset of hypersaline habitats, indicated that the species of *Natronomonas* are widely distributed in saline lakes and salterns as well as on saline soils. Species of this haloarchaeal genus can be considered as ubiquitous in intermediate to high salinity habitats.

## 1. Introduction

Haloarchaea are extremely halophilic archaea well adapted to hypersaline environments such as saline lakes, salterns, saline soils, or salted products (Ventosa, 2006; Oren, 2011). They are “*Euryarchaeota*” included into the class *Halobacteria*, with three orders: *Halobacteriales, Haloferacales*, and *Natrialbales,* that include three (*Halobacteriaceae*, *Halococcaceae*, and *Haloarculaceae*), two (*Haloferacaceae* and *Halorubraceae*), and one (*Natrialbaceae*) families, respectively, and more than 70 genera (Amoozegar et al., 2017; Oren et al., 2017; Parte et al., 2020).

The genus *Natronomonas* belongs to the family *Haloarculaceae,* and currently it includes six species: *Natronomonas pharaonis* (type species; Soliman and Trüper, 1982; Kamekura et al., 1997), *Natronomonas moolapensis* (Burns et al., 2009)*, Natronomonas gomsonensis* (Kim et al., 2013), *Natronomonas halophila* (Yin et al., 2020), *Natronomonas salina* (Yin et al., 2020), and *Natronomonas salsuginis* (Durán-Viseras et al., 2020). The most peculiar feature of the genus *Natronomonas* is that it comprises the first alkaliphilic and extremely halophilic archaeon so far described (*Nmn. pharaonis*), while the rest of the species of this genus are non-alkaliphilic. Although only six species have been described to date, several studies have shown that they are found in a variety of environments, such as hypersaline and alkaline lakes or soils, salterns or sediments, supporting the widespread distribution of this genus (Oren and Ventosa, 2017; Minegishi and Kamekura, 2018).

The members of the genus *Natronomonas* are characterized by a diverse cell morphology: short rods or pleomorphic shapes (coccoid, flat, and tetragonal shapes). They are motile and Gram-stain-negative. In solid culture media they produce red pigmented colonies. They are aerobic microorganisms and have a chemoorganotrophic metabolism. They are obligately halophilic (requiring at least 15% [w/v] NaCl for growth and growing optimally at 20–25% [w/v] NaCl). Alkaliphilic species grow at the pH range 8.0 to 11.0 (optimal growth at pH 8.5–9.0), but neutrophilic species grow at pH 5.5–8.5, and optimally at pH 7.0–7.5 (Minegishi and Kamekura, 2018). Membrane polar lipids have been shown to be a relevant feature for the taxonomic characterization of haloarchaea (Torreblanca et al., 1986; Oren et al., 2009; de la Haba et al., 2018). All members of the genus *Natronomonas* have phosphatidylglycerol and phosphatidylglycerol phosphate methyl ester. However, unidentified phospholipids or glycolipids may exist. The polar lipid profile of the haloalkaliphilic species *Nmn. pharaonis* is different from those of the neutrophilic species, without membrane glycolipids, and thus, it has been suggested that this species could be placed into a separated genus from the neutrophilic species of this genus (Minegishi and Kamekura, 2018).

Our studies on the prokaryotic diversity of several hypersaline environments of South-West Spain permitted us to isolate a new strain, designated as F2-12^T^, from the brine of a pond of one of those salterns. The aim of this paper is firstly to elucidate the phylogenomic and metabolic structure of the genus *Natronomonas*, based on current genomic-based analysis as well as phenotypic features, to clarify if *Nmn. pharaonis* and the remaining neutrophilic species of the genus should be separated into two different genera. Besides, we describe the taxonomic features of the new archaeon F2-12^T^ which we propose as a new species of the genus *Natronomonas*, *Natronomonas aquatica* sp. nov. Finally, we have carried out an in-depth analysis, based on metagenomics fragment recruitments, to show the ecological distribution and abundance of species of the genus *Natronomonas* in different hypersaline habitats.

## 2. Materials and methods

### 2.1. Isolation and culture of the new haloarchaeal strain

Strain F2-12^T^ was isolated from the brine of a crystallizer pond of a marine saltern located in Isla Cristina, Huelva, South-West Spain by Durán-Viseras (2020). It was isolated in pure culture on R2A medium supplemented with 25% (w/v) seawater salt solution. The composition of this salt mixture is (g l^−1^): NaCl, 195; MgCl_2_·6H_2_O, 32.5; MgSO_4_·7H_2_O, 50.8; CaCl_2_, 0.83; KCl, 5.0; NaHCO_3_, 0.21; NaBr, 0.58 (Durán-Viseras, 2020). The pH of the medium was adjusted to 7.5 with 1 M KOH. For the preparation of the solid media, purified agar was added at 1.8% (w/v). The culture was routinely incubated aerobically at 37°C for 14 days, both in liquid and solid medium. For comparative purposes, the type strains of species of the genus *Natronomonas*, *Natronomonas pharaonis* DSM 2160^T^, *Natronomonas gomsonensis* JCM 17867^T^, *Natronomonas moolapensis* CECT 7526^T^, and *Natronomonas salsuginis* F20-122^T^ were used as culture collection reference strains.

### 2.2. DNA extraction, purification, and sequencing

The DNA extraction and purification were carried out by the method described by Marmur (1961). DNA quantification and purity were determined by fluorometry (Qubit 3.0 Fluorometer, Thermofisher Scientific, USA) and spectrophotometry (NanoDrop One, Thermofisher Scientific, USA), respectively. The 16S rRNA and *rpoB’* genes were amplified by PCR using the primers ArchF (TTC CGG TTG ATC CTG CCG GA) and ArchR (GGT TAC CTT GTT ACG ACT T) as well as rpoBF (TGT AAA ACG ACG GCC AGT TCG AAG AGC CGG ACG ACA TGG) and rpoBR (CAG GAA ACA GCT ATG ACC GGT CAG CAC CTG BAC CGG NCC), respectively (Durán-Viseras, 2020). The PCR products were sequenced using the Sanger method by StabVida (Caparica, Portugal), and primers 16RB36 (GGA CTA CCA GGG TAT CTA) and 16RD34 (GGT CTC GCT CGT TGC CTG) were also used in order to obtain the complete sequence of 16S rRNA gene. The draft genome sequence of strain F2-12^T^ was also determined in this study by Illumina NovaSeq 2×150 PE (Novogene Co., United Kingdom).

### 2.3. Phylogenetic analyses

The 16S rRNA and *rpoB’* gene sequences of the new isolate, strain F2-12^T^, were analyzed, corrected, and assembled with ChromasPro version 1.5 (Technelysium Pty Ltd.). The 16S rRNA gene sequence was compared with EzBioCloud database.^1^ Using ARB software package (Ludwig et al., 2004) and LTPs_106_SSU database, alignments and phylogenetic trees were generated by three different methods: maximum-parsimony (Fitch, 1971), neighbor-joining (Saitou and Nei, 1987) and maximum-likelihood (Felsenstein, 1981) algorithms. A bootstrap analysis (1,000 pseudo-replicates) was performed to evaluate the robustness of the phylogenetic trees (Felsenstein, 1985). The *rpoB’* gene sequence was compared with other sequences available in NCBI database by BLAST search (http://www.ncbi.nlm.nih.gov/BLAST/; Altschul et al., 1990). The alignment and phylogenetic trees were obtained with BioEdit 3.3.19.0 version (Hall, 1999) and MEGA 6.11 software (Tamura et al., 2013),^2^ respectively.

The 16S rRNA and *rpoB’* gene sequences of strain F2-12^T^ were deposited in GenBank/EMBL/DDBJ, under the accession numbers MZ318646 and MZ327708, respectively.

### 2.4. Genome assembly, annotation, and determination of genomic parameters

The raw reads resulting from genome of strain F2-12^T^ were quality checked with FastQC 0.11.9 version (Andrews, 2010) and further trimmed (settings: qtrim = rl trimq = 18) and screened for adaptor and vector contamination (settings: k = 21 tbo ordered cardinality) using BBDuk from BBTools suite (Bushnell, 2021). Filtered reads were assembled with Spades v.3.12.0 (settings: --isolate -k 21, 33, 55, 77, 99, 127; Bankevich et al., 2012) and the quality of the assembly was quantified with QUAST v.2.3 (Gurevich et al., 2013). Finally, genome completeness and contamination were estimated with CheckM v.1.0.5 software (settings: lineage_wf --reduced_tree; Parks et al., 2015).

The genome sequence of strain F2-12^T^ was initially annotated using Prokka (default settings) and further following the NCBI Prokaryotic Genome Annotation Pipeline (PGAP; Tatusova et al., 2016) and was deposited in GenBank/EMBL/DDBJ, under the accession number GCA_024449025.1.

Several genomic indexes were used for circumscribing species and genera. The Orthologous Average Nucleotide Identity (OrthoANI) was determined with OAT v.0.93.1 (default settings; Lee et al., 2016), the digital DNA–DNA hybridization (dDDH) was calculated by the Genome-to-Genome Distance Calculator (GGDC) v.2.1 (Meier-Kolthoff et al., 2013) website, using BLAST+ as the local alignment tool and the formula 2 for distance calculations (Auch et al., 2019) and the Average Amino acid Identity (AAI) was calculated by CompareM (default settings; https://github.com/dparks1134/CompareM).

### 2.5. Phylogenomic comparative analysis

The phylogenomic comparative analysis was carried out using the genomes of the type strains of species of *Natronomonas*, as well as that of strain F2-12^T^ and other genomes of related haloarchaeal species available from GenBank database. The features and the accession numbers of the genomes of the type strains of the species of the genus *Natronomonas* used in this study are shown in Table 1. The quality of the genome sequences used in this study was in agreement with the recommended minimal standards for the use of genome data for the taxonomy of prokaryotes (Chun et al., 2018).

**Table 1:** General features of the genome sequences of *Natronomonas aquatica* F2-12^T^ and the type strains of the species of the genus *Natronomonas* used in this study.

Feature	**1**	**2**	**3**	**4**	**5**	**6**	**7**
Size (Mb)	3.21	2.90	2.91	2.75	3.21	3.75	3.38
Contigs	82	12	1	3	1	1	2
Genome coverage	508.3x	352.1x	60.1x	5.8x	100.0x	100.0x	100.0x
G + C (mol%)	62.7	63.2	64.5	63.1	64.4	67.5	64
N50 (bp)	84,628	675,769	2,912,573	2,595,221	3,211,682	3,746,575	1,800,000
Total genes	3,333	3,010	2,922	2,864	3,413	3,921	3,612
Protein coding genes	3,189	2,863	2,806	2,785	3,347	3,809	3,435
rRNA	3	3	3	3	3	3	3
tRNA	47	45	45	46	45	45	73
Assembly accession number	GCA_024449025.1	GCA_005239135.1	GCA_000591055.1	GCA_000026045.1	GCA_013391085.1	GCA_013391105.1	GCA_013391635.1

**1**, *Natronomonas aquatica* F2-12^T^, **2**, *Natronomonas salsuginis* F20-122^T^, **3**, *Natronomonas moolapensis* 8.8.11^T^, **4**, *Natronomonas pharaonis* DSM 2160^T^, **5,** *Natronomonas halophila* C90^T^, **6,** *Natronomonas salina* YPL13^T^, **7,** *Natronomonas gomsonensis* JCM 17867^T^.

To determine the core-genome, the Enveomics tool (Rodríguez-R and Konstantinidis, 2016) was used. To identify clusters of orthologous genes (OGs), we carried out an all-versus-all BLASTp search based on the translated protein-coding gene sequences of the type strains of species of *Natronomonas* and of strain F2-12^T^ as well as those of other genera of the family *Haloarculaceae* available in databases. The OGs shared among all taxa and present in a single copy per genome were selected for further analysis. They were aligned with MUSCLE v. 3.8.31 (Edgar, 2002) and subsequently concatenated. An approximately maximum-likelihood phylogenomic tree was obtained using FastTree v. 2.1.9 (Price et al., 2010) with the JTT replacement matrix (Jones et al., 1992) under the CAT approximation (single rate for each site) with 20 rate categories. Local support values were determined with the Shimodaira–Hasegawa test (Shimodaira and Hasegawa, 1999). An alternative phylogenomic tree based on selected 53 markers as recommended by the Genome Taxonomy Database (GTDB) was constructed using GTDB-Tk v. 2.1.0+ (Chaumeil et al., 2022) using the reference database R207_v2.

### 2.6. Phenotypic characterization

Phenotypic tests were determined for all species of the genus *Natronomonas* and they were carried out following the proposed minimal standards for the description of novel taxa in the class *Halobacteria* (Oren et al., 1997). Morphology and motility were studied by light optical under a phase-contrast microscope (Olympus BX41), in cultures incubated for 14 days at 37°C. The range and optimal growth conditions of salinity, pH, and temperature were determined on liquid medium R2A 25%, with the same composition but only modifying each of these factors. The medium R2A was supplemented with 0.9, 3, 5, 10, 15, 20, 25, and 30% (w/v) seawater salt solution (see section 2.1) to determine the growth under different salinity conditions. The growth at different pH values was determined in this medium supplemented with suitable buffers (MOPS, pH 6.0–7.0; Tris, pH 7.5–8.5; CHES, pH 9.0–10.0) to maintain stable the pH values to 6.0, 6.5, 7.0, 7.5, 8.0, 8.5, 9.0, and 10.0. The growth at 4, 15, 20, 25, 30, 37, 40, 45, 50, and 55°C permitted to determine the range and optimal growth at different temperatures.

Anaerobic growth was determined using R2A 25% plates supplemented with 1% L-arginine, 1% dimethyl sulfoxide (DMSO), and 1% KNO_3_, respectively, that were inoculated and incubated at 37°C in an anaerobic chamber (Oxoid) for 21 days. All biochemical tests were carried out in R2A 25% medium, pH 7.5 and 37°C. Catalase activity was observed by adding 3% (w/v) H_2_O_2_ to a colony of strain F2-12^T^ (Cowan and Steel, 1993). Oxidase activity was determined by adding 1% (w/v) tetramethyl-p-phenylenediamine (Kovács, 1956). Hydrolysis of starch, gelatin, aesculin or Tween 80 were determined as previously described (Durán-Viseras et al., 2020). Indole production was tested as described by Kovács (1928). Methyl red and Voges-Proskauer tests were performed by two methods (Werkman, 1930; Cowan and Steel, 1993). Nitrate and nitrite reduction was determined by using sulfanilic acid and α-naphthylamine (Skerman, 1967). The formation of H_2_S was determined as described by Ventosa et al. (1982). Acid production from carbohydrates was determined by adding a solution of the carbohydrate (1%, w/v, final carbohydrate concentration) to the basal medium with phenol red and supplemented with 0.05% (w/v) of yeast extract. Finally, the utilization of different compounds as sole carbon and energy source was determined by adding a filtered-sterilized solution of 1% (w/v) of different carbohydrates, alcohols, organic acids or amino acids to the medium SW25 supplemented with 0.05% (w/v) of yeast extract (Ventosa et al., 1982).

### 2.7. Chemotaxonomic analysis

The polar lipids of *Natronomonas moolapensis* CECT 7526^T^, *Natronomonas salsuginis* F20-122^T^, *Natronomonas gomsonensis* JCM 17867^T^ and strain F2-12^T^ were determined from cells cultured in medium R2A 25% at 37°C for 14 days, and the biomass from *Natronomonas pharaonis* CECT 4578^T^ was obtained in the alkaline medium (Soliman and Trüper, 1982) at 37°C for 14 days. The species *Halobacterium salinarum* DSM 3754^T^ and *Halorubrum saccharovorum* DSM 1137^T^ were used as reference for the determination of the polar lipids profiles. The total polar lipids were extracted as follows: the cell biomass was washed by adding 25% (w/v) NaCl sterile solution and centrifuged for 1 min at 6,000 *g*; the pellet was then resuspended in 0.8 ml of 25% (w/v) NaCl solution until obtaining a cell suspension. To 0.8 ml of cell suspension, 2 ml of methanol and 1 ml of chloroform were added to create a monophasic mixture. After gently mixing by inversion for 1 h and later centrifuged for 1 min at 6,000 *g*, the supernatant was collected from the colorless pellet. The supernatant in monophase was disrupted adding 500 μl of KCl 0.2 M and 1 ml of chloroform, followed by centrifugation during 1 min at 6,000 *g*. A bilayer phase was formed and the lower pigmented phase corresponding to the chloroform fraction was recovered and reduced to ~500 μl by evaporation under a fume hood or vacuum concentrator. The extract was transferred to a weighted empty glass vial of 2 ml, dried, weighted and stored at −20°C. To perform the chromatography, the total lipid extract was dissolved in a final concentration of 100 mg ml^−1^. A volume of 10 μl of total lipid extract (100 mg ml^−1^) were analyzed by high-performance thin layer chromatography (HPTLC), using HPTLC silica gel 60 plates crystal back (10 × 20 cm; Merck art. 5626); the plates were developed in the solvent system chloroform/methanol/90% (v/v) acetic acid (65:4:35) as previously described (Angelini et al., 2012). To detect all polar lipids, the plate was sprayed with 5% (v/v) sulfuric acid in water and charred by heating at 160°C.

### 2.8. Metagenomic fragment recruitment analyses

In order to determine the presence in different saline habitats of the six species of the genus *Natronomonas* and strain F2-12^T^, fragment recruitments with different environmental metagenomic datasets were performed. The genome contigs were concatenated and then all the rRNA gene sequences obtained were masked. BLASTn search (with the cut-offs: alignment length ≥30 nt, e-value ≤1 × 10^−5^, identity >95%) was used to align the metagenomic quality-filtered shotgun reads against the concatenated contigs of the type strains of all six species of the genus *Natronomonas*, as well as those of strain F2-12^T^. Recruitment plot representations were performed in R using the library “Hmisc.”

## 3. Results and discussion

### 3.1. The genus *Natronomonas* is a phylogenomically coherent taxonomic group

**Figure 1:**
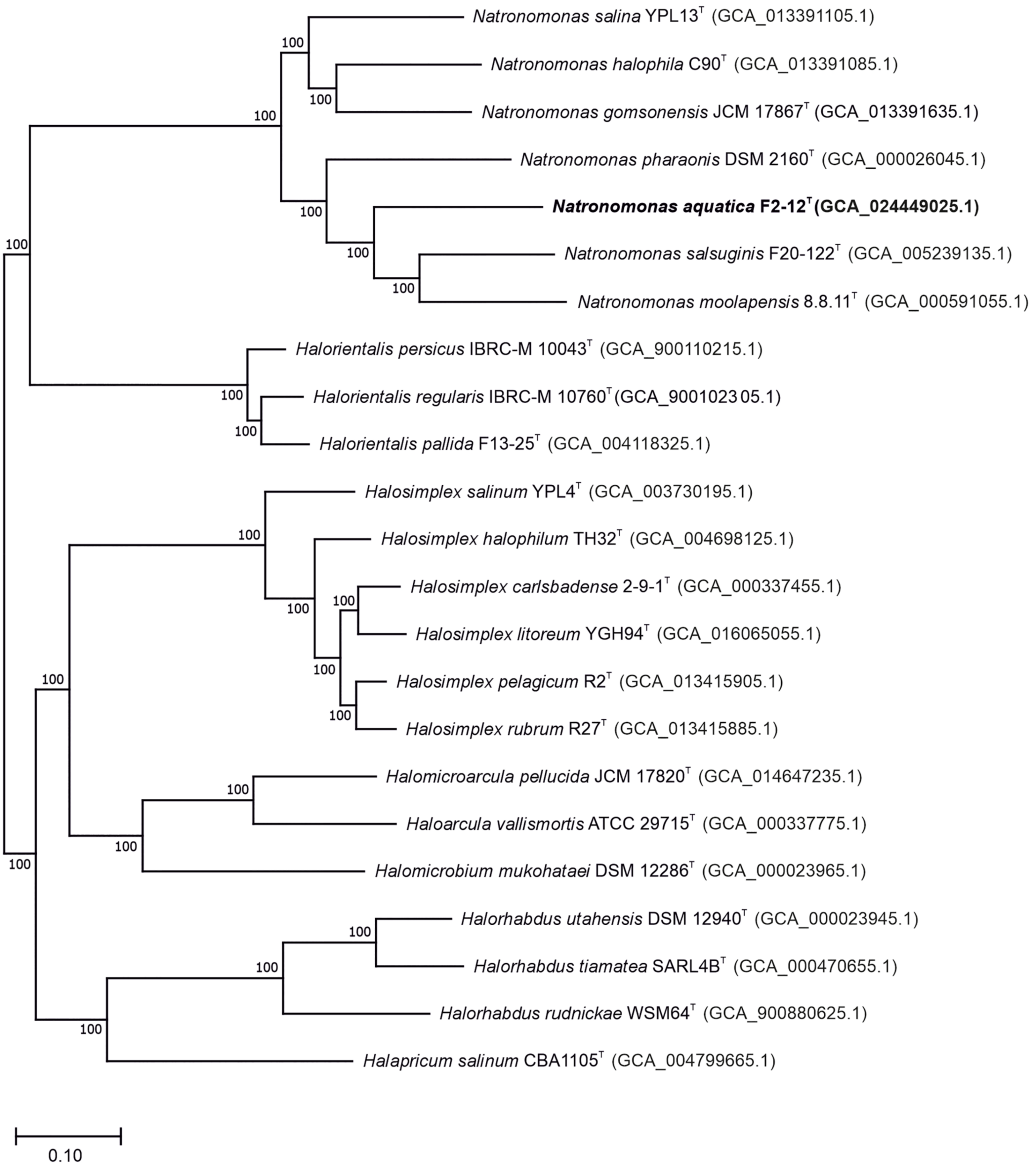
Approximately maximum-likelihood phylogenomic tree reconstruction based on the translated core orthologous genes of members of the genus *Natronomonas*, including strain F2-12^T^ and related species. This tree was obtained after the alignment and concatenation of the translated sequences of 870 shared orthologous single-copy genes of these genomes. Bootstrap values higher than 70% are indicated at branch points. Bar, 0.1 substitutions per nucleotide position.

Previous studies have suggested that the type species of the genus *Natronomonas*, *Nmn. pharaonis*, possesses, in fact, substantial differences as to be considered as a member of a separated genus from the remaining five species of *Natronomonas* (Minegishi and Kamekura, 2018). Those phenotypic differences are, essentially, the alkaliphilic behavior of *Nmn. pharaonis* versus the neutrophilic nature of the other five species, and the lack of glycolipids in the polar lipid profile of the haloalkaliphilic species *Nmn. pharaonis*. In order to shed light on this matter, a genome-based phylogeny was performed including all the validly described species names of the genus *Natronomonas* and closest relatives. This phylogenomic tree was obtained after the alignment and concatenation of the translated sequences of 870 core, orthologous, single-copy genes from the genomes under study (Figure 1). As it can be observed, all the species of the genus *Natronomonas*, including the conflicting species *Nmn. pharaonis*, formed a robust branch (100% bootstraps) that demonstrates that they constitute a monophyletic group of species. This study was complemented with the phylogenomic tree based on the comparison of the 53 concatenated conserved single-copy proteins recommended by the GTDB (Supplementary Figure S1), which also showed the monophyletic topology of the species of the genus *Natronomonas*, including *Nmn. pharaonis* and the new strain F2-12^T^.

**Figure 2:**
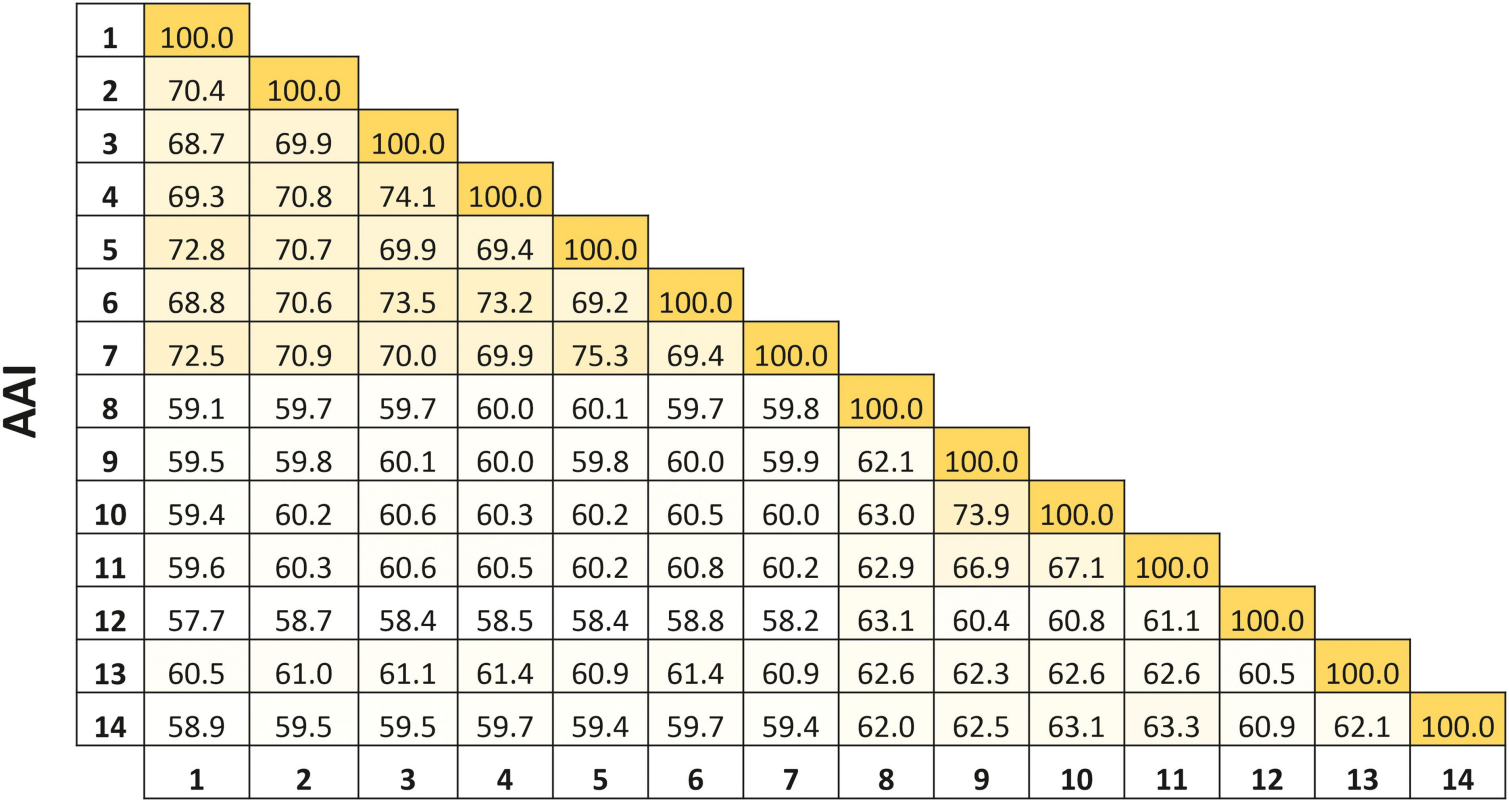
Average Amino acid Identity (AAI) percentages among the species of the genus *Natronomonas*, including strain F2-12^T^, and other related haloarchaeal genera. Strains: 1, *Natronomonas aquatica* F2-12^T^ (GCA_024449025.1); 2, *Natronomonas pharaonis* DSM 2160^T^ (GCA_000026045.1); 3, *Natronomonas gomsonensis* JCM 17867^T^ (GCA_013391635.1); 4, *Natronomonas halophila* C90^T^ (GCA_013391085.1); 5, *Natronomonas moolapensis* 8.8.11^T^ (GCA_000591055.1); 6, *Natronomonas salina* YPL13^T^ (GCA_013391105.1); 7; *Natronomonas salsuginis* F20-122^T^ (GCA_005239135.1); 8, *Halapricum salinum* CBA1105^T^ (GCA_004799665.1); 9, *Haloarcula vallismortis* ATCC 29715^T^ (GCA_000337775.1); 10, *Halomicroarcula pellucida* JCM 17820^T^ (GCA_014647235.1); 11, *Halomicrobium mukohataei* DSM 12286^T^ (GCA_000023965.1); 12, *Halorhabdus utahensis* DSM 12940^T^ (GCA_000023945.1); 13, *Halorientalis regularis* IBRC-M 10760^T^ (GCA_9001023 05.1); 14, *Halosimplex carlsbadense* 2-9-1^T^ (GCA_000337455.1).

On the other hand, the percentages of AAI among all species of *Natronomonas* were 68.7–75.3%, while the values between the species of *Natronomonas* and the other species of related haloarchaeal genera were equal to or lower than 61.4%. These percentages show unequivocally that all species of *Natronomonas* constitute a coherent genus, clearly separated from the other related genera (Figure 2). Thus, even considering that the species *Nmn. pharaonis* shows some differential phenotypic data, such as the optimal pH and range supporting growth or a different polar lipid profile than the other species of *Natronomonas*, there is no reason to taxonomically separate it from the remaining species of the genus *Natronomonas*.

### 3.2. A putative new species of the genus *Natronomonas*

During our studies on the culturable diversity of a Isla Cristina saltern, located in Huelva, Spain, the strain F2-12^T^ was isolated (Durán-Viseras, 2020). The almost-complete 16S rRNA gene sequence of this strain was amplified, sequenced, and analyzed (1,400 bp). The results generated by the EzBioCloud tool indicated that strain F2-12^T^ is a member of *Natronomonas,* showing the highest percentages of identity with the type strains of the species *Natronomonas moolapensis* 8.8.11^T^ (98.0%), *Natronomonas salsuginis* F20-122^T^ (97.3%), *Natronomonas pharaonis* DSM 2160^T^ (96.8%), *Natronomonas salina* YPL13^T^ (96.8%), *Natronomonas halophila* C90^T^ (96.7%) and *Natronomonas gomsonensis* SA3^T^ (95.8%), while the species of other related genera, such as *Halocatena* or *Salinirubellus,* showed percentages of similarity lower than 93.8%. The percentages of similarity between strain F2-12^T^ and all species of *Natronomonas* are lower than 98.7%, considered the cutoff delineation of prokaryotic species (Konstantinidis et al., 2017).

**Figure 3:**
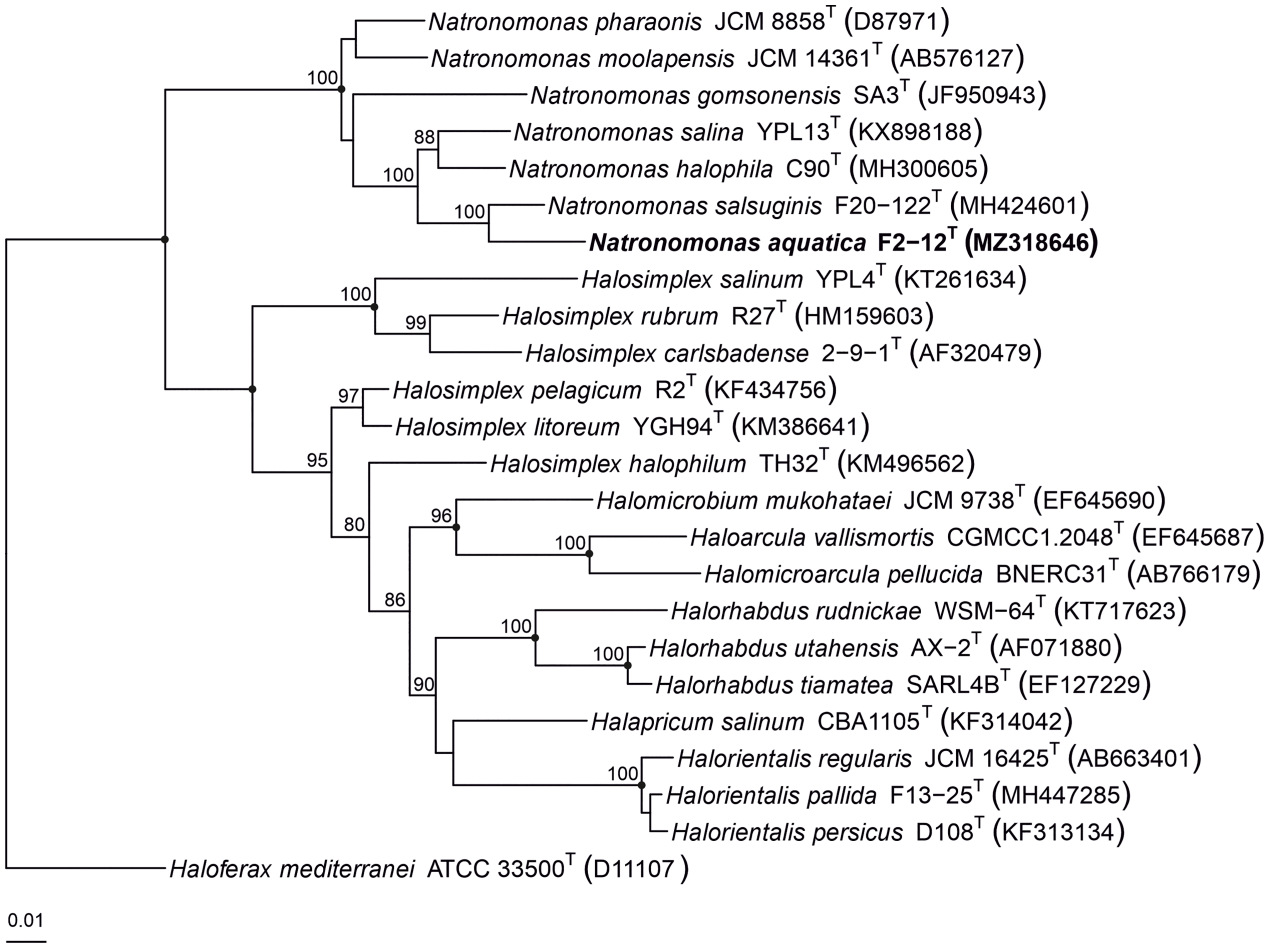
Maximum-parsimony phylogenetic tree based on the comparison of the 16S rRNA gene sequences showing the relationship between strain F2-12^T^, the species of the genus *Natronomonas* and other related haloarchaeal genera. The sequence accession numbers are shown in parentheses. Bootstrap values equal or higher than 70% are indicated above the nodes. Black circles indicate that the corresponding nodes were also obtained in the trees generated with the maximum-likelihood and neighbor-joining algorithms. *Haloferax mediterranei* ATCC 33500^T^ was used as outgroup. Bar, 0.01 substitutions per nucleotide position.

The 16S rRNA phylogenetic tree, constructed by the maximum-parsimony algorithm (Figure 3), showed that strain F2-12^T^ clustered with the species of the genus *Natronomonas,* but it constituted an independent branch. The phylogenetic position of strain F2-12^T^ was also confirmed in trees obtained using the maximum-likelihood and neighbor-joining algorithms. These results suggest that strain F2-12^T^ could be a new member of the genus *Natronomonas.*

**Figure 4:**
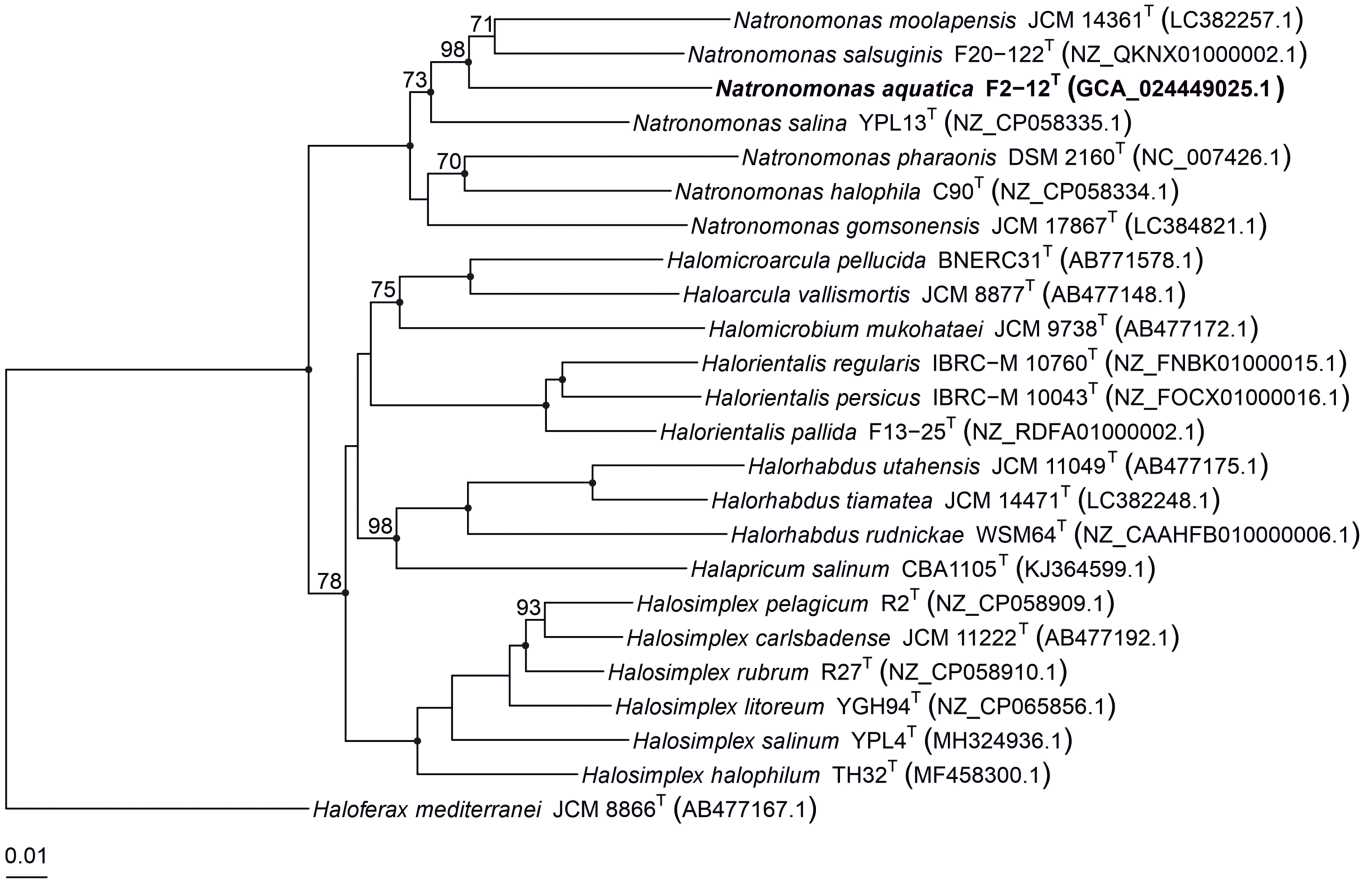
Maximum-parsimony phylogenetic tree based on the comparison of *rpoB*’ gene sequences showing the phylogenetic relationship between strain F2-12^T^, the species of the genus *Natronomonas* and other related haloarchaeal genera. The sequences accession numbers are shown in parentheses. Bootstrap values equal or higher than 70% are indicated above the nodes. Black circles indicate that the corresponding nodes were also obtained in the trees generated with the maximum-likelihood and neighbor-joining algorithms. *Haloferax mediterranei* ATCC 33500^T^ was used as outgroup. Bar, 0.01 substitutions per nucleotide position.

Several copies of the 16S rRNA gene with divergent sequences have been reported in species of haloarchaea, such as *Halosimplex*, *Haloarcula, Halomicrobium*, or *Halorientalis* (Vreeland et al., 2002; Boucher et al., 2004; Cui et al., 2009; Sun et al., 2013; Durán-Viseras et al., 2019). In order to avoid the limitations caused by the intragenomic heterogeneity among haloarchaeal 16S rRNA genes, it has been claimed the use of the *rpoB’* gene as an alternative phylogenetic marker in haloarchaea (Walsh et al., 2004; Enache et al., 2007; Minegishi et al., 2010). Thus, we also determined the partial *rpoB’* sequence (527 bp) of strain F2-12^T^ and used it for comparative studies between this strain and the already described species of *Natronomonas*. The analysis showed that strain F2-12^T^ was also related to species of the genus *Natronomonas*, showing percentages of similarity between 90.5 and 87.1% for the species of *Natronomonas* and lower percentages regarding to other haloarchaeal genera. A maximum-parsimony *rpoB’*-based phylogenetic tree was also obtained (Figure 4). This tree shows that strain F2-12^T^ belongs to the genus *Natronomonas*, but it is far enough away from species of this genus, and also supports the possibility that it could constitute a new species. On the other hand, both 16S rRNA and *rpoB’* gene-based phylogenetic trees show that all species of *Natronomonas* cluster together and formed a separate phylogenetic group with respect to species of the related haloarchaeal genera investigated, also supporting our aforementioned conclusion that no arrangements are necessary in the genus *Natronomonas*.

### 3.3. Genome-based analyses confirm the new species of *Natronomonas*

The draft genome sequence of strain F2-12^T^ was successfully assembled in 82 contigs, with a sequencing depth of 508.3x of the entire genome. The genome size of this strain was 3,214,353 bp and the DNA G + C content was 62.7 mol%. The range of G + C content for species of *Natronomonas* is from 63.1 to 67.5 mol%, with an intermediate value of 63.1 mol% for the type species of the genus, *Nmn. pharaonis*. The genome size ranged from 2.75 to 3.75 Mb, which are within those described for species of haloarchaea. Additional genomic characteristics are shown in Table 1. The comparison of this genome and those of the type strains of the species of *Natronomonas* and other closely related haloarchaea permitted us the reconstruction of the phylogenomic core-genome tree (Figure 1), as stated before. Strain F2-12^T^ belongs to the same clade of *Natronomonas moolapensis* 8.8.11^T^, *Natronomonas salsuginis* F20-122^T^, *Natronomonas pharaonis* DSM 2160^T^, *Natronomonas salina* YPL13^T^, *Natronomonas gomsonensis* JCM 17867^T^ and *Natronomonas halophila* C90^T^, but clustered in a different branch, separated from the rest of species of *Natronomonas*, with a bootstrap percentage of 100%, reinforcing the hypothesis of being a new species of the genus *Natronomonas*.

**Figure 5:**
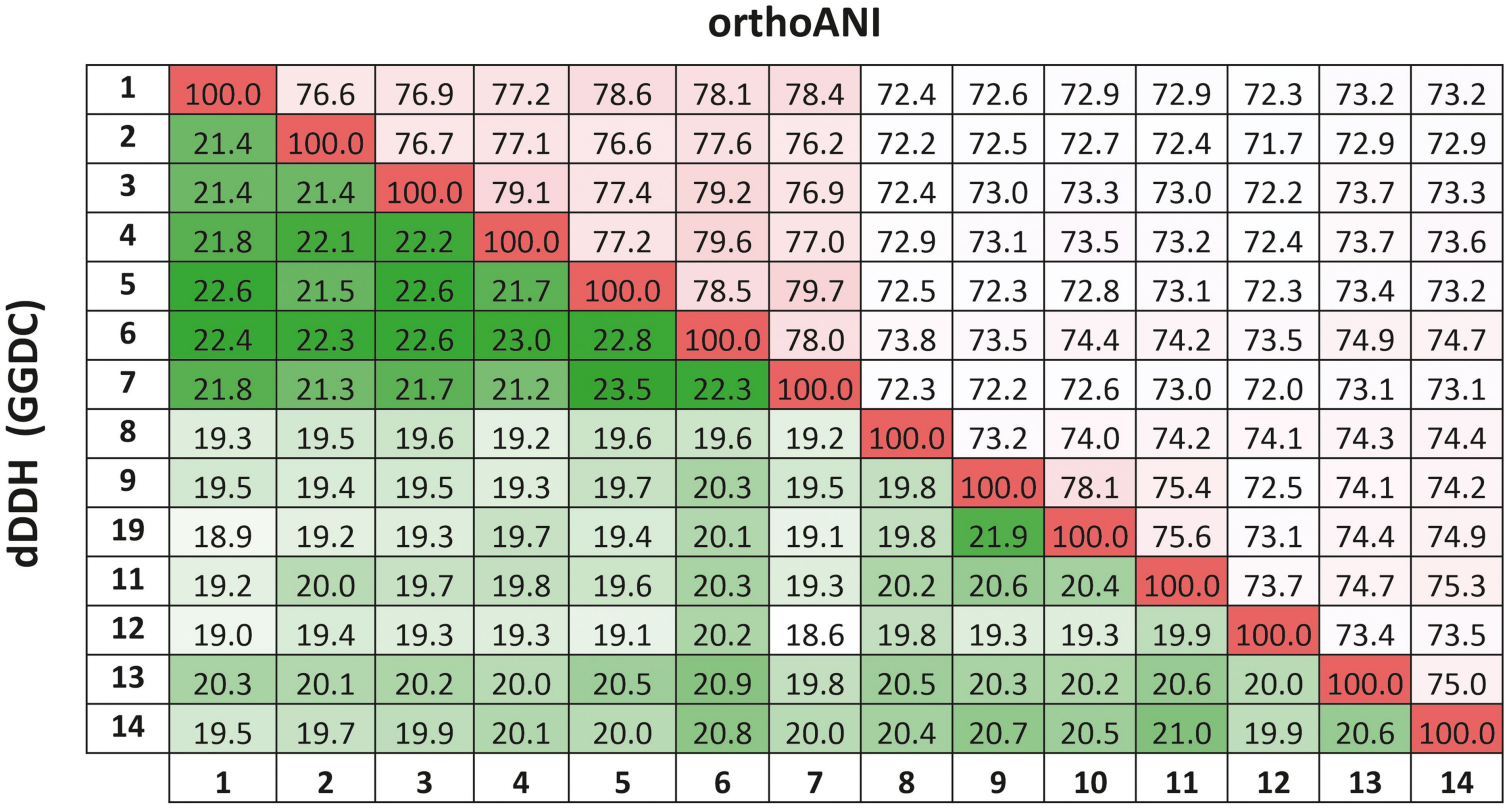
Average Nucleotide Identities (orthoANI) and digital DNA–DNA hybridization (dDDH) calculated by the Genome-to-Genome Distance Calculator (GGDC) percentages of strain F2-12^T^, the species of the genus *Natronomonas* and other related genera. Strains: 1, *Natronomonas aquatica* F2-12^T^ (GCA_024449025.1); 2, *Natronomonas pharaonis* DSM 2160^T^ (GCA_000026045.1); 3, *Natronomonas gomsonensis* JCM 17867^T^ (GCA_013391635.1); 4, *Natronomonas halophila* C90^T^ (GCA_013391085.1); 5, *Natronomonas moolapensis* 8.8.11^T^ (GCA_000591055.1); 6, *Natronomonas salina* YPL13^T^ (GCA_013391105.1); 7; *Natronomonas salsuginis* F20-122^T^ (GCA_005239135.1); 8, *Halapricum salinum* CBA1105^T^ (GCA_004799665.1); 9, *Haloarcula vallismortis* ATCC 29715^T^ (GCA_000337775.1); 10, *Halomicroarcula pellucida* JCM 17820^T^ (GCA_014647235.1); 11, *Halomicrobium mukohataei* DSM 12286^T^ (GCA_000023965.1); 12, *Halorhabdus utahensis* DSM 12940^T^ (GCA_000023945.1); 13, *Halorientalis regularis* IBRC-M 10760^T^ (GCA_9001023 05.1); 14, *Halosimplex carlsbadense* 2-9-1^T^ (GCA_000337455.1).

The previous findings were confirmed by calculating the overall genome relatedness indexes, namely OrthoANI (Average Nucleotide Identity), dDDH (digital DNA–DNA hybridization), and AAI (Average Amino acid Identity), which are currently used as genomic thresholds for delineation of new prokaryotic taxa (Meier-Kolthoff et al., 2013; Lee et al., 2016; Auch et al., 2019).The percentages of OrthoANI between the strain F2-12^T^ and the species of the genus *Natronomonas* ranged from 76.2 to 79.6% (Figure 5). These values are lower than the threshold percentage currently accepted for species delineation (95%; Konstantinidis et al., 2017), confirming that strain F2-12^T^ should be considered as a different species of the genus *Natronomonas*. On the other hand, percentages of dDDH higher or equal to 70% indicate that the strains can constitute the same species, while values lower than 70% show that strains belong to different prokaryotic species (Konstantinidis and Tiedje, 2004; Goris et al., 2007; Kim et al., 2014). Figure 5 shows that the percentages of dDDH were equal or lower than 23.5% between strain F2-12^T^ and the species of the genus *Natronomonas*, supporting that this strain constitutes a separate species of this genus. Finally, AAI was calculated to confirm that strain F2-12^T^ is well assigned to the genus *Natronomonas*. The AAI percentages of strain F2-12^T^ with respect to the species of *Natronomonas* were in the range of 68.8 to 72.8, and 60.5% or lower with respect to species of other related haloarchaeal genera (Figure 2). Since the AAI threshold established for species assigned to the same genus is 65% (Goris et al., 2007; Konstantinidis et al., 2017), we can confirm that strain F2-12^T^ certainly belong to the genus *Natronomonas*.

### 3.4. Phenotypic characterization of the new proposed species

For the phenotypic characterization, we compared strain F2-12^T^ with the most closely related species under the same laboratory conditions, as recommended by Oren et al. (1997). Cells of strain F2-12^T^ were motile rods of different sizes (0.3 μm width by 1–3 μm length), and produced circular, pink pigmented colonies with 1 mm in diameter on R2A 25% medium after 14 days of incubation at 37°C (Table 2). Strain F2-12^T^ is an extremely halophilic archaeon. It is not able to grow in media without NaCl. It can grow at 15–30% (w/v) total salts and its optimal growth is at 25% (w/v) salts. It is a neutrophilic haloarchaeon, growing at a pH range of 7.0–8.5, with optimal growth at pH 7.5–8.0. Concerning the temperature, strain F2-12^T^ is able to grow from 20 to 50°C and optimally at 37°C (Table 2). Catalase and oxidase activity are negative. Able to reduce nitrate and nitrite and to produce acids from D-glucose, as well as to utilize D-glucose, glycerol and pyruvate as sole carbon and energy source. The results for other biochemical and nutritional tests are described in Table 2, showing the differential characteristics with respect to the most closely related species of *Natronomonas*.

**Table 2:** Differential characteristics of *Natronomonas aquatica* F2-12^T^, *Natronomonas salsuginis* F20-122^T^, *Natronomonas moolapensis* CECT 7526^T^, *Natronomonas pharaonis* DSM 2160^T^, *Natronomonas gomsonensis* SA3^T^, *Natronomonas halophila* C90^T^, and *Natronomonas salina* YPL13^T^.

Characteristics	**1**	**2**	**3**	**4**	**5**	**6**	**7**
Morphology	Rods	Coccoid^a^	Pleomorphic^b^	Rods^c^	Coccoid^d^	Pleomorphic^e^	Pleomorphic^e^
Motility	+	−	+	+	+^d^	+^e^	+^e^
Cell size (μm)	0.3 × 1–3	1.0 × 1.2–2.5^a^	0.7 × 1.7^b^	0.8 × 1–3^c^	0.8–0.9 × 1.2^d^	ND	ND
Colony size (mm)	1	0.2–0.3	0.5–1.0	0.5	1.0–2.0^d^	ND	ND
Colony pigmentation	Pink	Pink	Red	Red	Red^d^	Red^e^	Red^e^
NaCl range (optimum) (%, w/v)	15–30(25)	10–30(25)^a^	14–36(18–20)^b^	12–30(20)^c^	18–30(24)^d^	5–28(25)^e^	5–28(20)^e^
Temperature range for growth (optimum) (°C)	20–50(37)	25–50(37)^a^	25–45(37–45)^b^	20–50(45)^c^	20–45(40)^d^	30–60(40)^e^	25–50(37)^e^
pH range (optimum)	7.0–8.5(7.5–8.0)	6.5–9.0(8.0)^a^	5.5–8.5(7.0–7.5)^b^	8.0–11.00(8.5–9.0)^c^	5.5–8.0(7.0)^d^	6.5–9.5(8.0)^e^	5.0–8.0(6.5)^e^
Oxidase	−	+	−	+	+^d^	−^e^	+^e^
Indole production	−	−	−	+	−^d^	−^e^	−^e^
Hydrolysis of gelatin	−	−	−	+	−^d^	−^e^	−^e^
Reduction of nitrate	+	+	+	−	−^d^	+^e^	+^e^
Reduction of nitrite	+	+	−	−	−^d^	+^e^	+^e^
Production of acids from carbohydrates:							
D-fructose	−	+	−	ND	+^d^	ND	ND
D-glucose	+	+	−	ND	+^d^	ND	ND
Utilization as sole carbon and energy source of:							
D-glucose	+	−	+	ND	+^d^	−^e^	−^e^
Glycerol	+	−	+	ND	+^d^	−^e^	−^e^
Fumarate	−	−	+	ND	+^d^	+^e^	+^e^
Pyruvate	+	−	+	ND	+^d^	+^e^	+^e^

Strains: **1**, *Natronomonas aquatica* F2-12^T^, **2**, *Natronomonas salsuginis* F20-122^T^, **3**, *Natronomonas moolapensis* CECT 7526^T^, **4**, *Natronomonas pharaonis* DSM 2160^T^, **5**, *Natronomonas gomsonensis* SA3^T^, **6**, *Natronomonas halophila* C90^T^, **7**, *Natronomonas salina* YPL13^T^. All data from this study, except ^a^Durán-Viseras et al. (2020), ^b^Burns et al. (2009), ^c^Soliman and Trüper (1982) and Burns et al. (2009), ^d^Kim et al. (2013) and ^e^Yin et al. (2020). +, Positive; −, negative; ND, not determined.

### 3.5. Chemotaxonomic characterization of the new proposed species

The total polar lipids of strain F2-12^T^ were extracted and compared with those from the closely related neighbors, *Natronomonas salsuginis* F20-122^T^, *Natronomonas moolapensis* CECT 7526^T^, and *Natronomonas pharaonis* CECT 4578^T^, as well as with the reference strains *Halorubrum saccharovorum* DSM 1137^T^ and *Halobacterium salinarum* DSM 3754^T^, which permitted an identification of the major polar lipids of the new isolate.

The HPTLC (Supplementary Figure S2) revealed that the polar lipids profile of strain F2-12^T^ consisted of phosphatidylglycerol (PG) and phosphatidylglycerol phosphate methyl ester (PGP-Me), both derived from C_20_C_20_ and C_20_C_25_ archaeol, and phosphatidylglycerol sulfate (PGS) as major polar lipids. Traces of biphosphatidylglycerol (BPG), minor phospholipids, and an unidentified glycolipid were also detected. The profile of polar lipids of the new strain F2-12^T^ shares the major polar lipids described for all species of the genus *Natronomonas,* except for the haloalkaliphilic species *Nmn. pharaonis* CECT 4578^T^, that presents a different lipid profile, possibly due to its adaptation to alkaline environments (Soliman and Trüper, 1982). This feature has already been determined for alkaliphilic species of other haloarchaeal genera. Although the lipid pattern of the haloarchaeal species may vary according to the culture conditions, the alkaliphilic species of *Halorubrum* lack phosphatidylglycerol sulfate and glycolipids, that are present on neutrophilic species of this genus (Oren et al., 2009; Corral et al., 2016; de la Haba et al., 2018). A similar situation has been reported for the haloalkaliphilic species *Halostagnicola alkaliphila* (Nagaoka et al., 2011) and *Halostagnicola bangensis* (Corral et al., 2015), in which glycolipids have not been detected, in contrast to the neutrophilic species of the genus *Halostagnicola* (Castillo et al., 2006). A recent study of the polar lipid composition of extremely haloalkaliphilic strains from hypersaline lakes (Bale et al., 2019) also showed the absence of glycolipids in these isolates, and determined the presence of new cardiolipins, that were related to glycocardiolipins previously described in two alkaliphilic species of haloarchaea (Angelini et al., 2012). It has been postulated that this feature may be characteristic of alkaliphilic strains growing at low Mg^2+^ concentrations (Bale et al., 2019).

### 3.6. Metabolism of the genus *Natronomonas*

Based on the genome annotation from representative species on the genus *Natronomonas* and strain F2-12^T^, the major metabolic pathways of this group of microorganisms were inferred. Regarding the carbohydrate metabolism, the complete pathways for glycolysis, gluconeogenesis citrate and glyoxylate cycles were identified along all analyzed genomes, confirming the heterotrophic metabolism for members of the genus *Natronomonas*.

Concerning the nitrogen metabolism, it was very diverse. Genes involved in assimilatory nitrate reduction (*nasAB* and *nirA*) were present in the genomes of *Nmn. pharaonis*, *Nmn. halophila*, and *Nmn. salina*, while this pathway was incomplete on the genomes analyzed for the other studied strains (*Nmn. moolapensis*, *Nmn. salsuginis*, strain F2-12^T^, and *Nmn. gomsonensis*) which exclusively presented the gene *nirA*. The species *Nmn. pharaonis* also exhibited a complete dissimilatory nitrate reduction pathway to ammonia, while *Nmn. salsuginis* and *Nmn. moolapensis* presented the *nirBD* gene involved in the same pathway for nitrite reduction to ammonia. Additionally, different genes involved in the denitrification pathway were found in the genomes of *Nmn. pharaonis*, *Nmn. gomsonensis*, *Nmn. halophila*, and *Nmn. salina*, indicating that some of the species of this genus could also carry out modular steps of denitrification.

On the other side, strain F2-12^T^ and *Nmn. salina* exhibited the almost complete assimilatory sulfate reduction pathway which converts inorganic sulfate to sulfide, which is further incorporated into carbon skeletons of amino acids to form cysteine. The complete pathways for the biosynthesis of the amino acids threonine, cysteine, valine, isoleucine, lysine, arginine, proline and tryptophan were encountered in all the genomes under study, while the leucine biosynthesis pathway was also found in strain F2-12^T^, *Nmn. gomsonensis*, *Nmn. halophila*, and *Nmn. salina* and the histidine biosynthesis pathway in *Nmn. gomsonensis*, *Nmn. halophila*, and *Nmn. salina*.

Overall, the current species of the genus *Natronomonas* and strain F2-12^T^ exhibited similar metabolic reconstructions, even considering the alkaliphilic nature of *Nmn. pharaonis* and the neutrophilic nature of other species of the genus, which further support the taxonomic coherence of this group of archaea as a single genus.

### 3.7. Ecological importance and environmental abundance of *Natronomonas*

**Figure 6:**
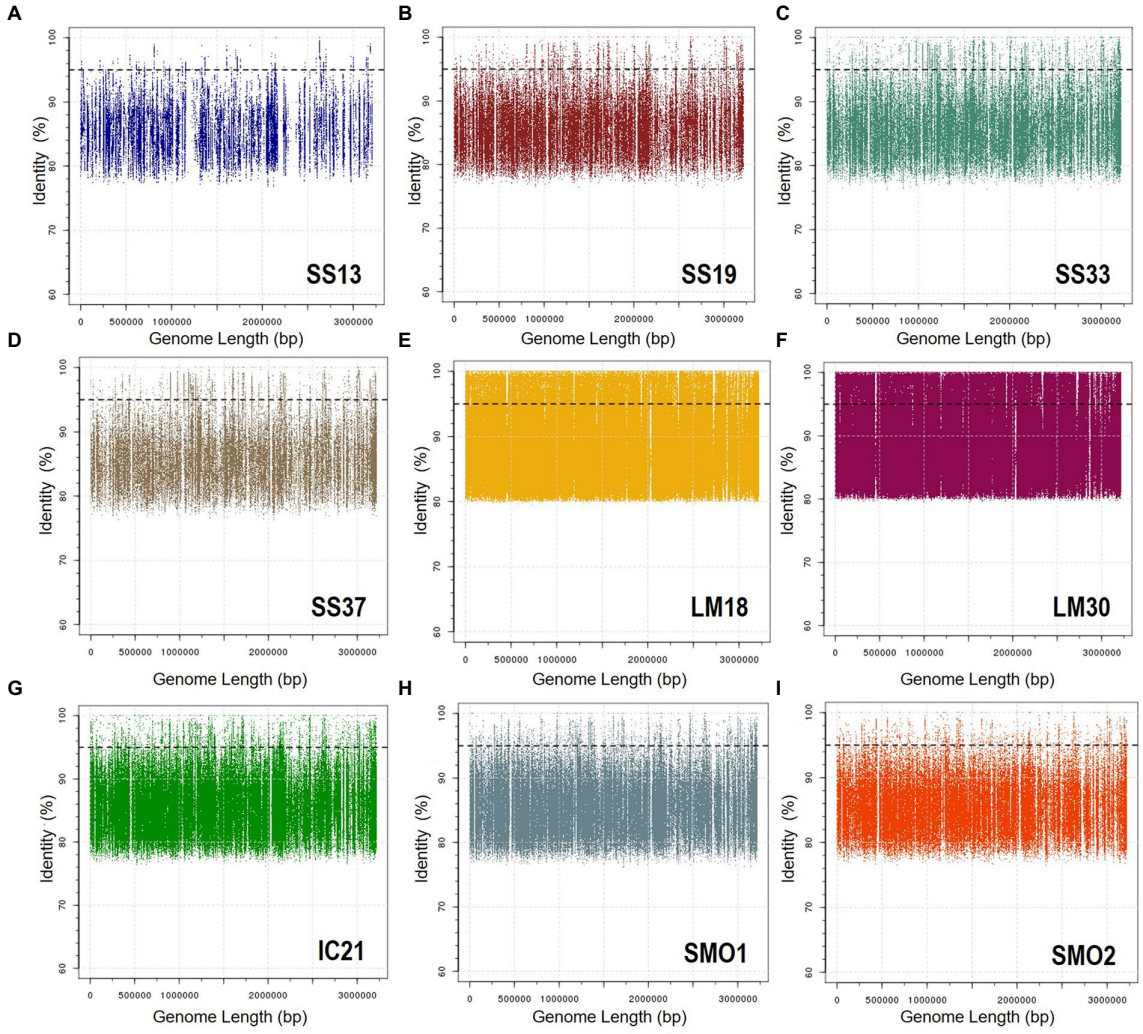
Recruitment plots of *Natronomonas aquatica* F2-12^T^ against different metagenomic datasets: **(A)** SS13, **(B)** SS19, **(C)** SS33, **(D)** SS37, **(E)** LM18, **(F)** LM30, **(G)** IC21, **(H)** SMO1, and **(I)** SMO2. In each panel the Y axis represents the identity percentage and X axis represents the genome length. The black dashed line shows the threshold for the presence of same species (95% identity). Abbreviations: SS13: metagenome from Santa Pola saltern (Spain) with 13% salinity (SRX328504; Fernández et al., 2014a), SS19: metagenome from Santa Pola saltern (Spain) with 19% salinity (SRX090228; Ghai et al., 2011), SS33: metagenome from Santa Pola saltern (Spain) with 33% salinity (SRX347883; Fernández et al., 2013), SS37: metagenome from Santa Pola saltern (Spain) with 37% salinity (SRX090229; Ghai et al., 2011), LM18: metagenome from Lake Meyghan (Iran) with 18% salinity (ERS1455390; Naghoni et al., 2017), LM30: metagenome from Lake Meyghan (Iran) with 30% salinity (ERS1455391; Naghoni et al., 2017), IC21: metagenome from Isla Cristina saltern (Spain) with 21% salinity (Fernández et al., 2014b,c), SMO1: metagenome from Odiel saltmarshes hypersaline soil, 24 mS/cm conductivity (SRR5753725; Vera-Gargallo and Ventosa, 2018), SMO2: metagenome from Odiel saltmarshes hypersaline soil, 54 mS/cm conductivity (SRR5753724; Vera-Gargallo and Ventosa, 2018).

To determine the presence of the new species of *Natronomonas*, *Nmn. aquatica* F2-12^T^, as well as of the other six species of the genus *Natronomonas* in several aquatic and terrestrial hypersaline environments, fragment recruitments with different environmental metagenomic datasets were performed. Figure 6 shows the recruitments of strain F2-12^T^ against nine metagenomic databases from Santa Pola salterns, Alicante, Spain (brines from ponds with salinities of 13, 19, 33, and 37%), Lake Meyghan in Iran (salinities of 18 and 30%), Isla Cristina saltern, Huelva, Spain (21% salinity) and hypersaline soils located in Odiel Saltmarshes, Huelva, Spain (conductivities of 24 and 54 mS/cm). In general, recruitments were evident in saline habitats with intermediate to high salinity, especially in the two metagenomes from Lake Meyghan and that from Isla Cristina saltern (hypersaline habitat from which this new species was isolated).

With respect to the other six species of the genus *Natronomonas*, the recruitment studies show that they are also more abundant in the Lake Meyghan (at intermediate and high salinities) in comparison to the other hypersaline environments tested (Supplementary Figures S3–S8). These results are consistent with previous data by Naghoni et al. (2017), indicating that the genus *Natronomonas* constituted one of the most dominant genera inhabiting that lake. It is noteworthy that they are also detected on the two metagenomic datasets from hypersaline soils, indicating that the members of the genus *Natronomonas* are ubiquitous in hypersaline habitats, being present in aquatic as well as in terrestrial hypersaline environments. Finally, the abundance of metagenomic reads recruited below 95% similarity might suggests the existence of additional species belonging to the genus *Natronomonas* present in these habitats.

## 4. Conclusion

The 16S rRNA gene, *rpoB’* gene, phylogenomic analysis, genomic indexes OrthoANI, dDDH and AAI, and metabolic analysis demonstrate that the current species of the genus *Natronomonas* constitute a coherent taxonomic group, even considering that the type species of the genus, *Nmn. pharaonis,* is a haloalkaliphilic archaeon that shows some specific phenotypic characteristics, such as a different polar lipid profile, probably due to its adaptive features to alkaline environments. The new strain F2-12^T^, isolated in pure culture from the pond of a saltern located in Isla Cristina, Spain, was studied and characterized using genomic and classical taxonomic methods. This study shows that strain F2-12^T^ constitutes a new species of the genus *Natronomonas*, and thus, we propose to name it as *Natronomonas aquatica* sp. nov. The formal description of this new species is given below. Finally, the metagenomic recruitment studies show that members of the genus *Natronomonas* are widely distributed on aquatic (salterns, lake) and also on terrestrial hypersaline habitats.

### Description of *Natronomonas aquatica* sp. nov.

*Natronomonas aquatica* (a.qua’ti.*ca.* L. fem. adj. *aquatica*, lives or grows in the water, aquatic, it is referred to the place where it was isolated).

Cells are Gram-stain-negative, motile rods with a size of 0.3 μm × 1–3 μm. Colonies are circular, pink pigmented and 1 mm in diameter on R2A medium supplemented with 25% salts after 14 days of incubation at 37°C. Chemoorganotrophic and aerobic. Extremely halophilic and neutrophilic archaeon, growing in media with 15–30% (w/v) NaCl and with an optimal growth at 25% (w/v) NaCl. Not able to grow in the absence of NaCl. The pH range for growth is 7.0–8.5 and the optimal growth is at pH 7.5–8.0. Able to grow from 20 to 50°C and optimally at 37°C. Does not grow anaerobically with potassium nitrate, L-arginine or DMSO. Catalase and oxidase activity are negative. Aesculin, gelatin, starch and Tween 80 are not hydrolyzed. Nitrate and nitrite are reduced, H_2_S is not produced from thiosulfate. Simmons’ citrate and Voges-Proskauer tests are negative. Methyl red test is positive. Indole is not produced from tryptophan. Acid is produced from D-glucose and D-ribose but not from D-amygdalin, D, L-ethionine, D-arabinose, arbutin, D-cellobiose, L-citrulline, D-fructose, D-galactose, glycerol, inulin, D-maltose, D-mannitol, D-mannose, D-melezitose, D-raffinose, D-sucrose, sorbitol, D-trehalose, L-xylitol, and D-xylose. The following compounds are used as sole source of carbon and energy: D-arabinose, D-glucose, glycerol, xylitol, L-alanine, glutamine, isoleucine and pyruvate, but not fructose, lactose, maltose, L-raffinose, sucrose, D-trehalose, D-xylose, salicin, butanol, D-mannitol, propanol, L-arginine, L-cysteine, L-methionine, L-glycine, L-lysine, valine, benzoate, citrate, formate, fumarate, propionate, valerate, hippurate, malate, and tartrate. The major polar lipids are phosphatidylglycerol (PG), phosphatidylglycerol phosphate methyl ester (PGP-Me), and phosphatidylglycerol sulfate (PGS). Traces of biphosphatidylglycerol (BPG) and other minor phospholipids and an unidentified glycolipid may also be present.

The type strain is F2-12^T^ (= CCM 9001^T^ = CECT 9970^T^ = JCM 33798^T^), and was isolated from a marine saltern located in Isla Cristina, Huelva, Spain. The DNA G + C content of the type strain F2-12^T^ is 62.7 mol%, as determined from its genome.

The GenBank/EMBL/DDBJ accession number for the 16S rRNA and *rpoB’* gene sequences of strain F2-12^T^ are MZ318646 and MZ327708, respectively, and that of the genome sequence is GCA_024449025.1.

## Data availability statement

The datasets presented in this study can be found in online repositories. The names of the repository/repositories and accession number(s) can be found below: https://www.ncbi.nlm.nih.gov/genbank/, MZ318646, MZ327708, and JAHLKM000000000.

## Author contributions

AD-V, CS-P, and AV did the conceptualization. AD-V performed the isolation of the strain. AG-R carried out the phenotypic characterization. AG-R and AD-V carried out the genomic analyses, RRH contributed to the phylogenomic analyses, PC to the polar lipids composition and CS-P to the recruitment experiments. AG-R, AD-V, CS-P, and AV prepared the draft manuscript and the tables and figures. AV and CS-P did the funding acquisition. All authors contributed to the article and approved the submitted version.

## Funding

This study was supported by grants PID2020-118136GB-I00 funded by MCIN/AEI/10.13039/501100011033 and by ESF Investing in your future and by Junta de Andalucía (Spain; P20_01066 and BIO-213), all including European (FEDER) funds. AG-R was the recipient of a predoctoral fellowship from the Spanish Ministry of Universities. AD-V was supported by a Margarita Salas postdoctoral research fellowship from the Spanish Ministry of Universities (financed by the European Union, under the NextGenerationEU funds). RRH was the recipient of a short-stay grant “Salvador Madariaga” from the Spanish Ministry of Science, Innovation and Universities (Programa Estatal de Promoción del Talento y su Empleabilidad en I + D + I, Subprograma Estatal de Movilidad, del Plan Estatal de Investigación Científica y Técnica y de Innovación 2017–2020; PRX21/00598).

## Conflict of interest

The authors declare that the research was conducted in the absence of any commercial or financial relationships that could be constructed as a potential conflict of interest.

## Publisher’s note

All claims expressed in this article are solely those of the authors and do not necessarily represent those of their affiliated organizations, or those of the publisher, the editors and the reviewers. Any product that may be evaluated in this article, or claim that may be made by its manufacturer, is not guaranteed or endorsed by the publisher.

## Footnotes

^1^ https://www.ezbiocloud.net/

^2^ https://doi.org/10.1093/molbev/msab120
