# Supplementary material for: Genomic-based phylogenetic and metabolic analyses of the genus Natronomonas, and description of Natronomonas aquatica sp. nov
Source: Front Microbiol. 2023 Jan 20;14:1109549. doi: 10.3389/fmicb.2023.1109549 (PMC9895928; doi:10.3389/fmicb.2023.1109549)
Supplement: Supplementary file 1 [file Data_Sheet_1.pdf]

Genomic-based phylogenetic and metabolic analyses of the genus *Natronomonas*, and description of *Natronomonas aquatica* sp. nov.

Alicia García-Roldán [1], Ana Durán-Viseras [1], Rafael R. de la Haba [1], Paulina Corral [2], Cristina Sánchez-Porro [1,*] and Antonio Ventosa [1,*]

[1]Department of Microbiology and Parasitology, Faculty of Pharmacy, University of Sevilla, 41012 Sevilla, Spain.
[2]Department of Biology, University of Naples Federico II, Naples, Italy.

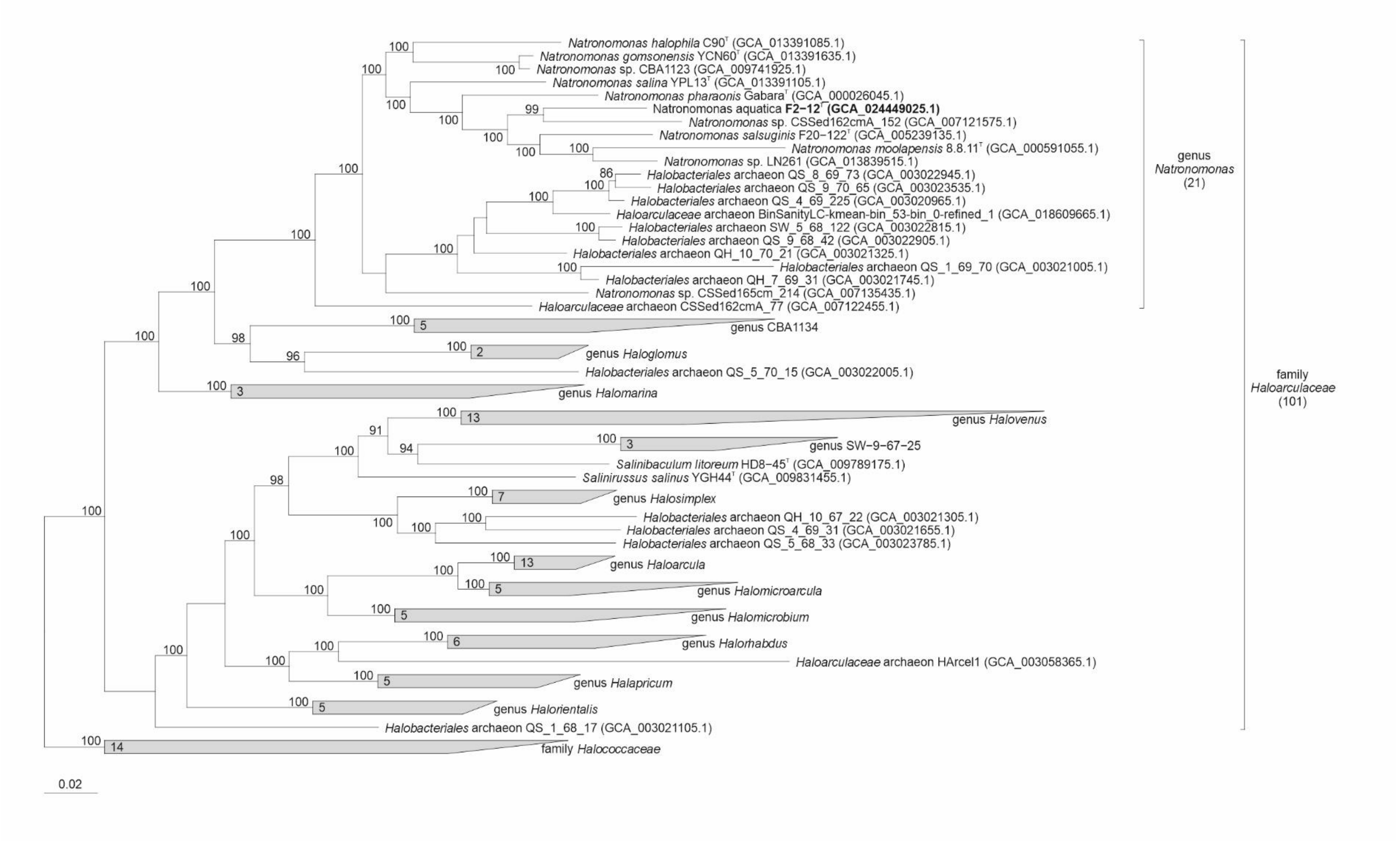

Figure S1. Approximately maximum-likelihood phylogenomic tree reconstruction based on 53 concatenated single copy marker proteins of members of the genus *Natronomonas*, including strain F2-12^T, related species of the family *Haloarculaceae* and other haloarchaea. Bootstrap values higher than 70% are indicated at branch point. Bar, 0.02 substitutions per nucleotide position.

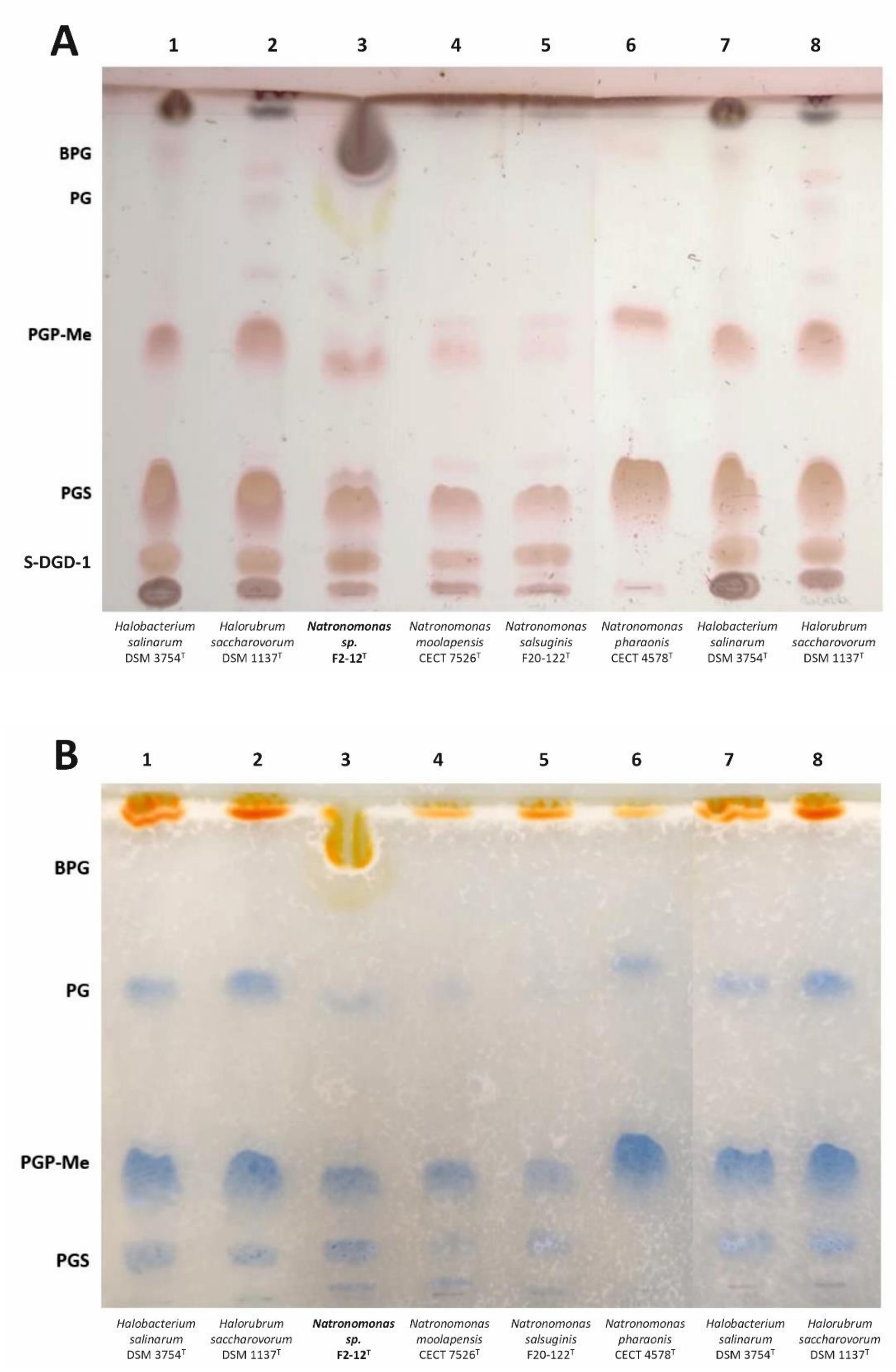

Figure S2. High performance thin layer chromatography (HPTLC) of polar lipids extracted from *Natronomonas* sp. F2-12^T, related species of the genus *Natronomonas* and other haloarchaeal species used as reference. A) The plate was revealed with sulfuric acid 5 % in water, followed charred by heating at 160 °C. B) The plate was revealed with molybdenum blue spray reagent. Lanes: 1, *Halobacterium salinarum* DSM 3754^T; 2, *Halorubrum saccharovorum* DSM 1137^T; 3, *Natronomonas* sp. F2-12^T; 4, *Natronomonas moolapensis* CECT 7526^T; 5, *Natronomonas salsuginis* F20-122^T; 6, *Natronomonas pharaonis* CECT 4578^T; 7, *Halobacterium salinarum* DSM 3754^T; 8, *Halorubrum saccharovorum* DSM 1137^T. **Abbreviations:** BPG, biphosphatidylglycerol; PG, phosphatidylglycerol; PGP-Me, phosphatidylglycerol phosphate methyl ester; PGS, phosphatidylglycerol sulfate; S-DGD-1, sulfated diglycosyl diether.

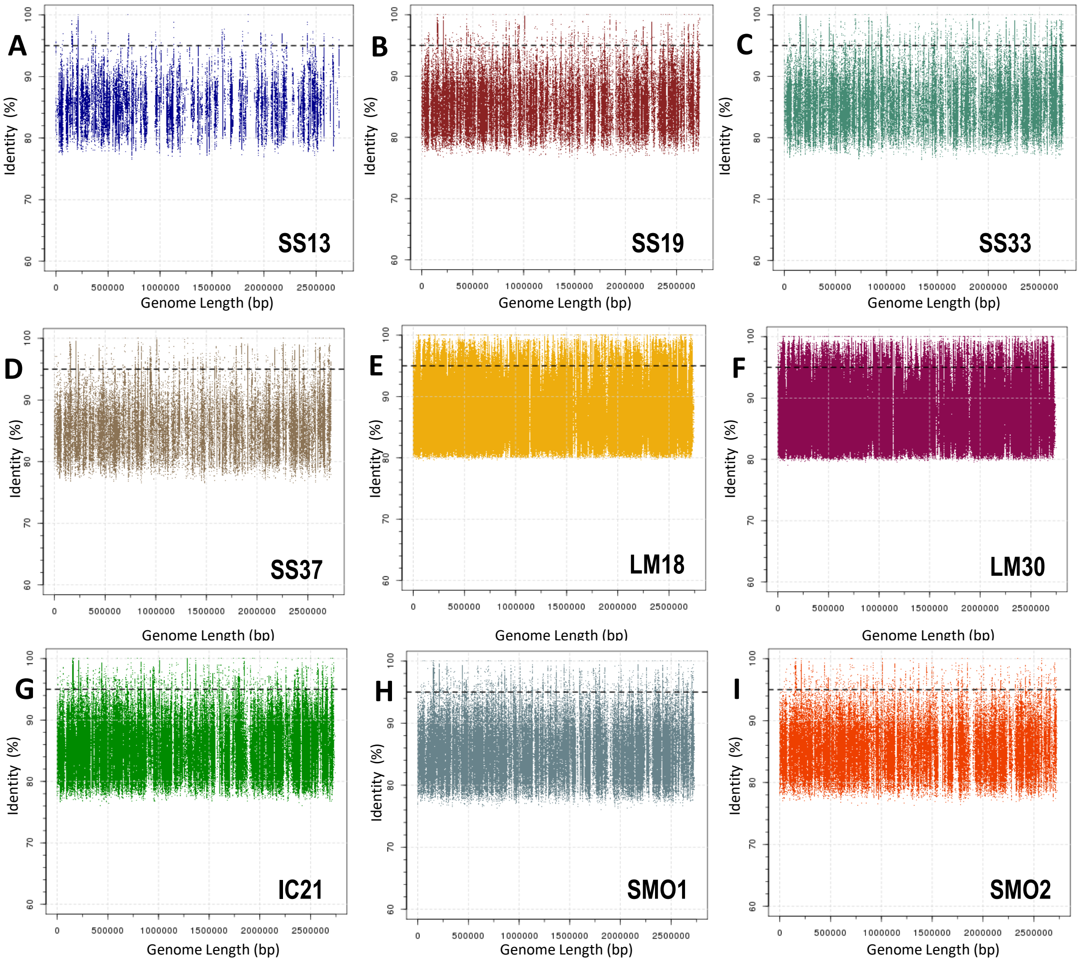

Figure S3. Recruitment plots of *Natronomonas pharaonis* DSM 2160ᵀ (GCA 000026045.1) against the metagenomic datasets: (A) SS13, (B) SS19, (C) SS33, (D) SS37, (E) LM18, (F) LM30, (G) IC21, (H) SMO1 and (I) SMO2. In each panel the Y axis represents the identity **percentage** and X axis represents the genome length. A restrictive cut-off 95% of nucleotide identity in at least 30 bp of the metagenomic read was used. The black dashed line shows the threshold for the presence of same species (95% identity). Abbreviations: SS13: metagenome from Santa Pola saltern (Spain) with 13% salinity (SRX328504) [1]; SS19: metagenome from Santa Pola saltern (Spain) with 19% salinity (SRX090228) [2]; SS33: metagenome from Santa Pola saltern (Spain) with 33% salinity (SRX347883) [3]; SS37: metagenome from Santa Pola saltern (Spain) with 37% salinity (SRX090229) [1]; LM18: metagenome from Lake Meyghan (Iran) with 18% salinity (ERS1455390) [4]; LM30: metagenome from Lake Meyghan (Iran) with 30% salinity (ERS1455391) [4]; IC21: metagenome from Isla Cristina saltern (Spain) with 2% salinity [5,6]; SMO1: metagenome from Odiel saltmarshes hypersaline soil, 24 mS/cm conductivity (SRR5753725) [7]; SMO2: metagenome from Odiel saltmarshes hypersaline soil, 54 mS/cm conductivity (SRR5753724) [7].

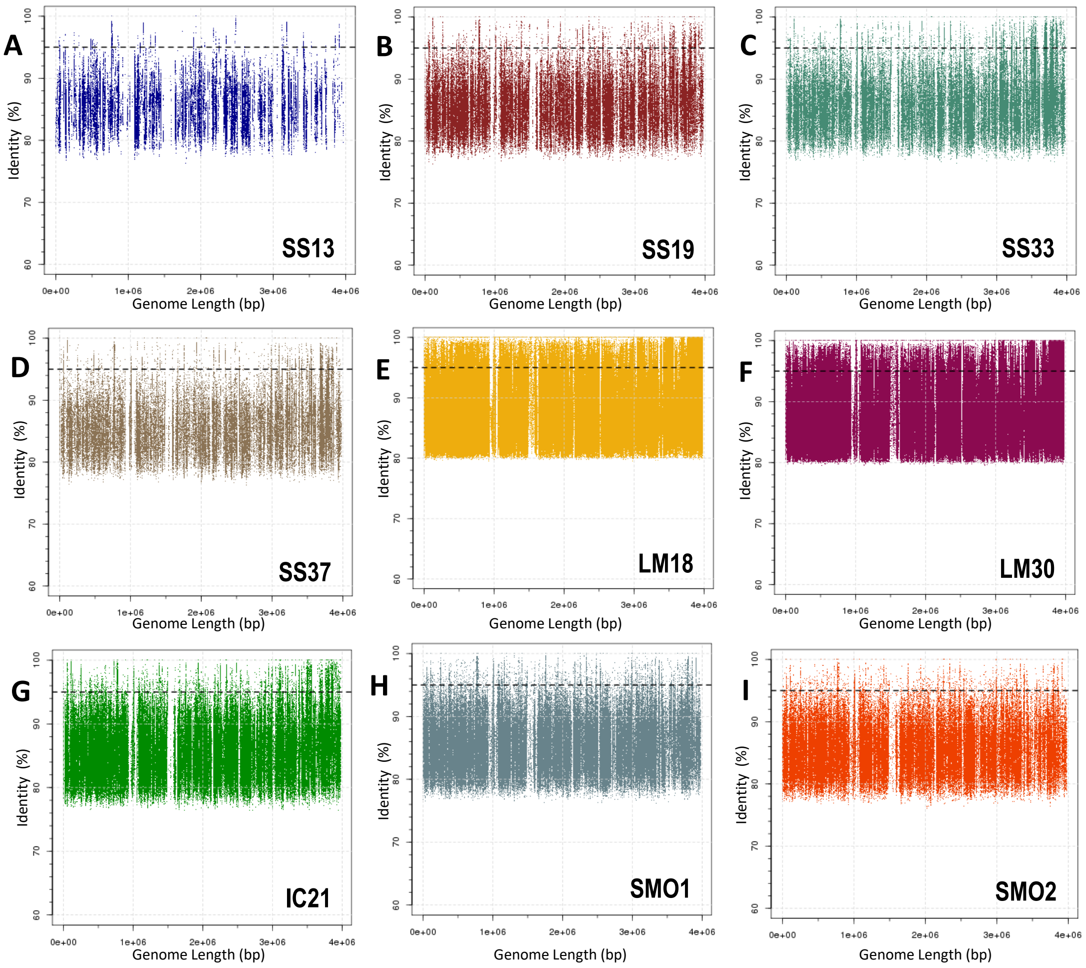

Figure S4. Recruitment plots of *Natronomonas gomsonensis* JCM 17867^{T} (GCA_013391635.1) against the metagenomic datasets: (A) SS13, (B) SS19, (C) SS33, (D) SS37, (E) LM18, (F) LM30, (G) IC21, (H) SMO1 and (I) SMO2. In each panel the Y axis represents the identity percentage and X axis represents the genome length. A restrictive cut-off 95% of nucleotide identity in at least 30 bp of the metagenomic read was used. The black dashed line shows the threshold for the presence of same species (95% identity). Abbreviations: SS13: metagenome from Santa Pola saltern (Spain) with 13% salinity (SRX328504) [1]; SS19: metagenome from Santa Pola saltern (Spain) with 19% salinity (SRX090228) [2]; SS33: metagenome from Santa Pola saltern (Spain) with 33% salinity (SRX347883) [3]; SS37: metagenome from Santa Pola saltern (Spain) with 37% salinity (SRX090229) [1]; LM18: metagenome from Lake Meyghan (Iran) with 18% salinity (ERS1455390) [4]; LM30: metagenome from Lake Meyghan (Iran) with 30% salinity (ERS1455391) [4]; IC21: metagenome from Isla Cristina saltern (Spain) with 21% salinity [5,6]; SMO1: metagenome from Odiel saltmarshes hypersaline soil, 24 mS/cm conductivity (SRR5753725) [7]; SMO2: metagenome from Odiel saltmarshes hypersaline soil, 54 mS/cm conductivity (SRR5753724) [7].

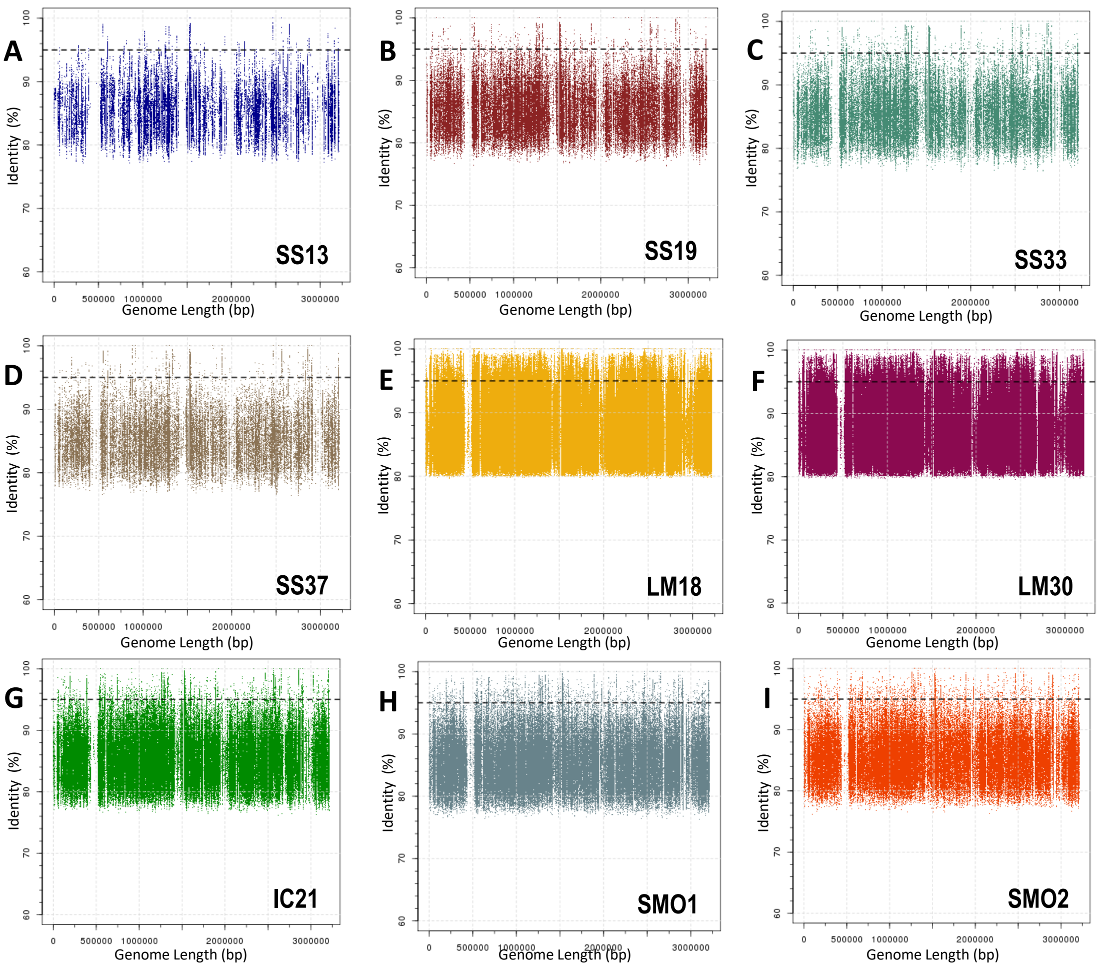

Figure S5. Recruitment plots of *Natronomonas halophila* C90^T (GCA 013391085.1) against the metagenomic datasets: (A) SS13, (B) SS19, (C) SS33, (D) SS37, (E) LM18, (F) LM30, (G) IC21, (H) SMO1 and (I) SMO2. In each panel the Y axis represents the identity percentage and X axis represents the genome length. A restrictive cut-off 95% of nucleotide identity in at least 30 bp of the metagenomic read was used. The black dashed line shows the threshold for the presence of same species (95% identity). Abbreviations: SS13: metagenome from Santa Pola saltern (Spain) with 13% salinity (SRX328504) [1]; SS19: metagenome from Santa Pola saltern (Spain) with 19% salinity (SRX090228) [2]; SS33: metagenome from Santa Pola saltern (Spain) with 33% salinity (SRX347883) [3]; SS37: metagenome from Santa Pola saltern (Spain) with 37% salinity (SRX090229) [1]; LM18: metagenome from Lake Meyghan (Iran) with 18% salinity (ERS1455390) [4]; LM30: metagenome from Lake Meyghan (Iran) with 30% salinity (ERS1455391) [4]; IC21: metagenome from Isla Cristina saltern (Spain) with 21% salinity [5,6]; SMO1: metagenome from Odiel saltmarshes hypersaline soil, 24 mS/cm conductivity (SRR5753725) [7]; SMO2: metagenome from Odiel saltmarshes hypersaline soil, 54 mS/cm conductivity (SRR5753724) [7].

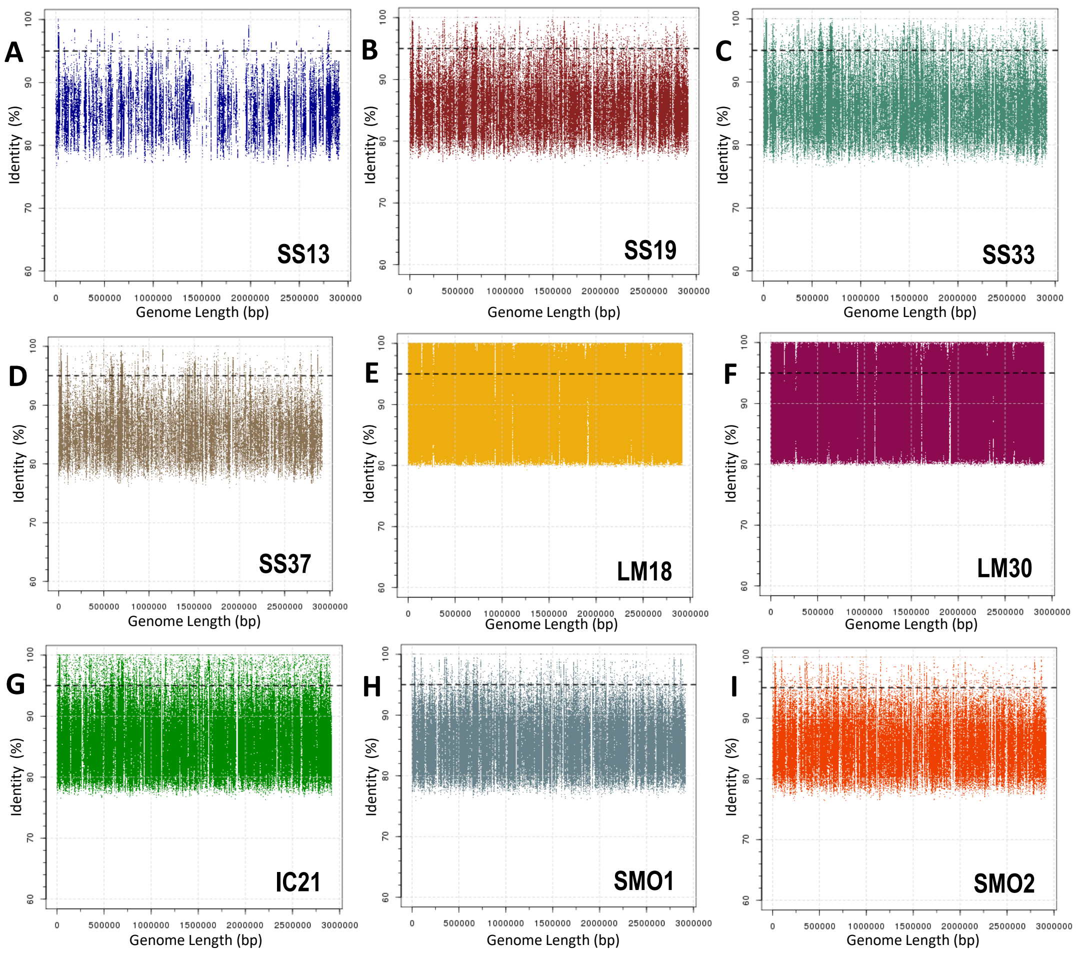

Figure S6. Recruitment plots of *Natronomonas moolapensis* 8.8.11^{T} (GCA 000591055.1) against the metagenomic datasets: (A) SS13, (B) SS19, (C) SS33, (D) SS37, (E) LM18, (F) LM30, (G) IC21, (H) SMO1 and (I) SMO2. In each panel the Y axis represents the identity percentage and X axis represents the genome length. A restrictive cut-off 95% of nucleotide identity in at least 30 bp of the metagenomic read was used. The black dashed line shows the threshold for the presence of same species (95% identity). Abbreviations: SS13: metagenome from Santa Pola saltern (Spain) with 13% salinity (SRX328504) [1]; SS19: metagenome from Santa Pola saltern (Spain) with 19% salinity (SRX090228) [2]; SS33: metagenome from Santa Pola saltern (Spain) with 33% salinity (SRX347883) [3]; SS37: metagenome from Santa Pola saltern (Spain) with 37% salinity (SRX090229) [1]; LM18: metagenome from Lake Meyghan (Iran) with 18% salinity (ERS1455390) [4]; LM30: Metagenome from Lake Meyghan (Iran) with 30% salinity (ERS1455391) [4]; IC21: metagenome from Isla Cristina saltern (Spain) with 21% salinity [5,6]; SMO1: Metagenome from Odiel saltmarshes hypersaline soil, 24 mS/cm conductivity (SRR5753725) [7]; SMO2: metagenome from Odiel saltmarshes hypersaline soil, 54 mS/cm conductivity (SRR5753724) [7].

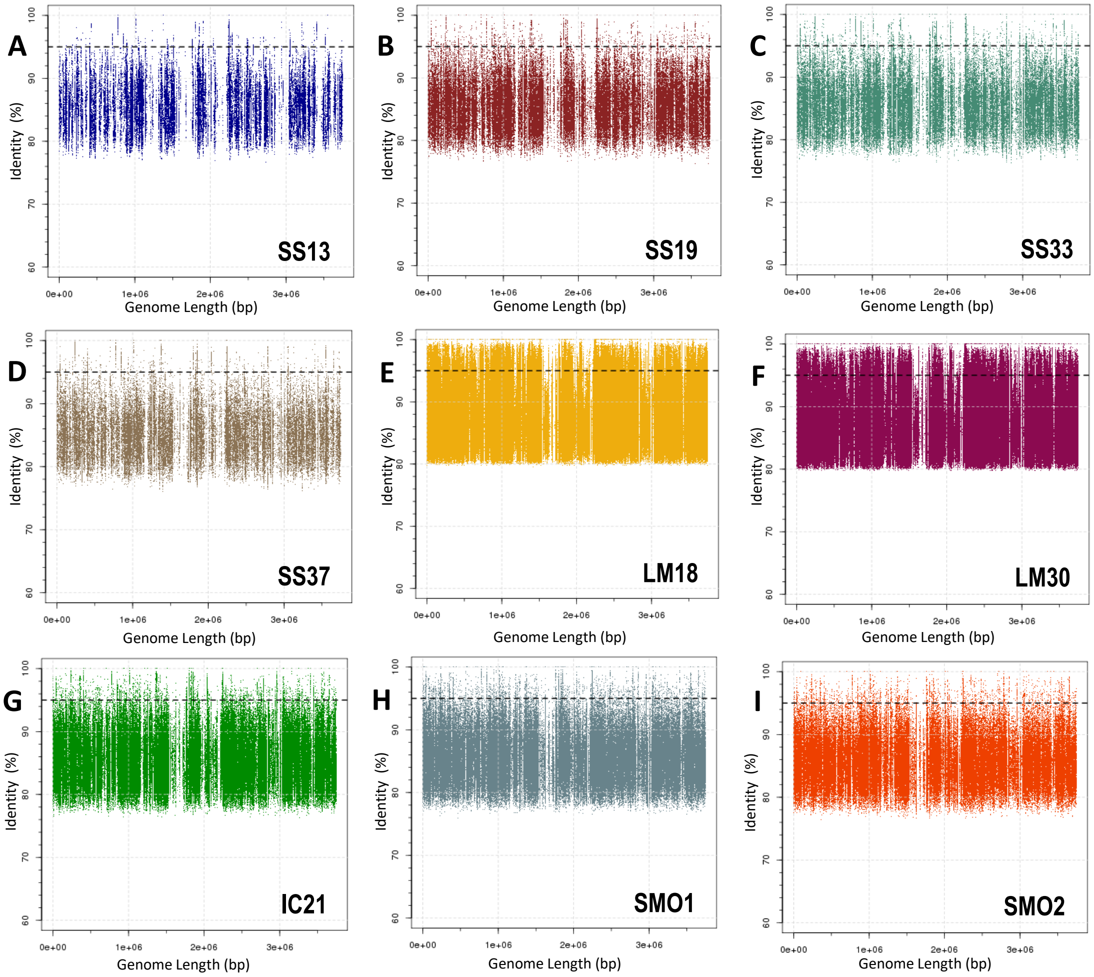

Figure S7. Recruitment plots of *Natronomonas salina* YPL13^T (GCA 013391105.1) against the metagenomic datasets: (A) SS13, (B) SS19, (C) SS33, (D) SS37, (E) LM18, (F) LM30, (G) IC21, (H) SMO1 and (I) SMO2. In each panel the Y axis represents the identity percentage and X axis represents the genome length. A restrictive cut-off 95% of nucleotide identity in at least 30 bp of the metagenomic read was used. The black dashed line shows the threshold for the presence of same species (95% identity). Abbreviations: SS13: metagenome from Santa Pola saltern (Spain) with 13% salinity (SRX328504) [1]; SS19: metagenome from Santa Pola saltern (Spain) with 19% salinity (SRX090228) [2]; SS33: metagenome from Santa Pola saltern (Spain) with 33% salinity (SRX347883) [3]; SS37: metagenome from Santa Pola saltern (Spain) with 37% salinity (SRX090229) [1]; LM18: metagenome from Lake Meyghan (Iran) with 18% salinity (ERS1455390) [4]; LM30: metagenome from Lake Meyghan (Iran) with 30% salinity (ERS1455391) [4]; IC21: metagenome from Isla Cristina saltern (Spain) with 21% salinity [5,6]; SMO1: metagenome from Odiel saltmarshes hypersaline soil, 24 mS/cm conductivity (SRR5753725) [7]; SMO2: metagenome from Odiel saltmarshes hypersaline soil, 54 mS/cm conductivity (SRR5753724) [7].

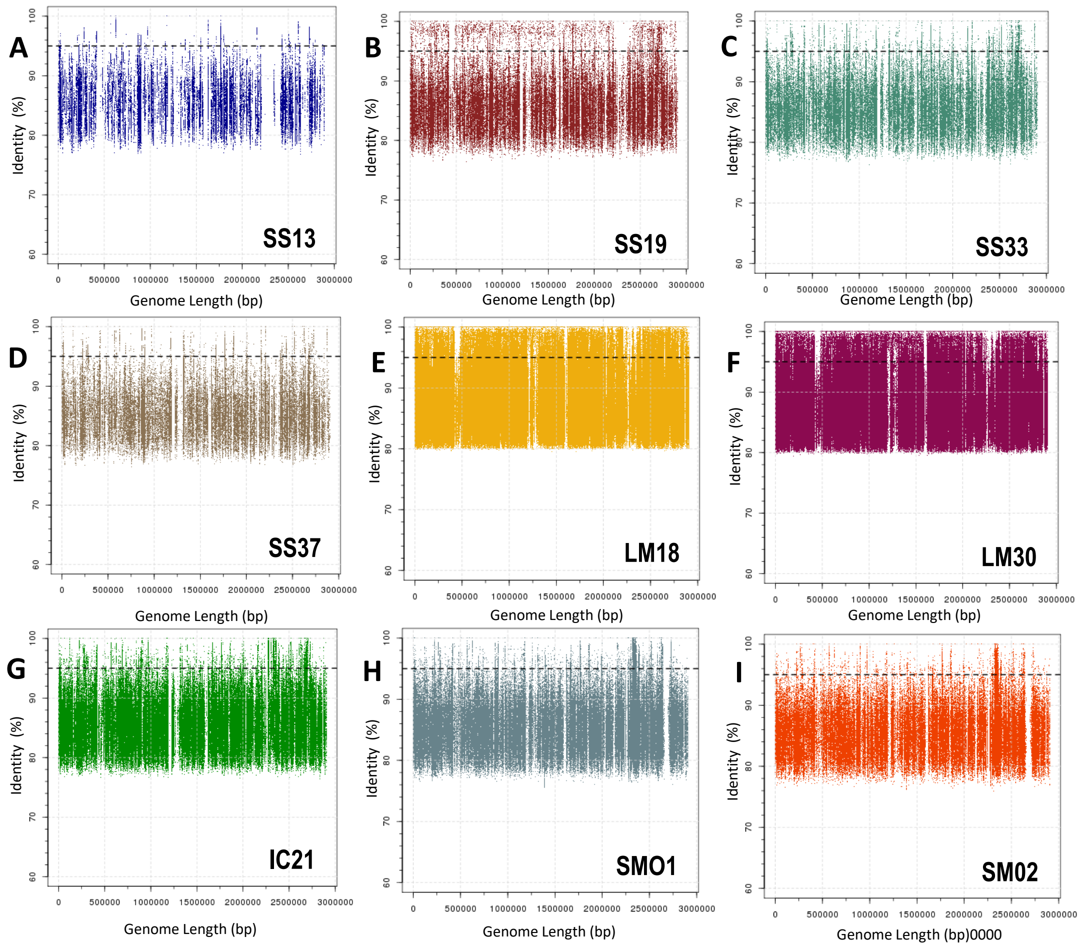

Figure S8. Recruitment plots of *Natronomonas salsuginis* F20-122^T (GCA 005239135.1) against the metagenomic datasets: (A) SS13, (B) SS19, (C) SS33, (D) SS37, (E) LM18, (F) LM30, (G) IC21, (H) SMO1 and (I) SMO2. In each panel the Y axis represents the identity percentage and X axis represents the genome length. A restrictive cut-off 95% of nucleotide identity in at least 30 bp of the metagenomic read was used. The black dashed line shows the threshold for the presence of same species (95% identity). Abbreviations: SS13: metagenome from Santa Pola saltern (Spain) with 13 % salinity (SRX328504) [1]; SS19: metagenome from Santa Pola saltern (Spain) with 19% salinity (SRX090228) [2]; SS33: metagenome from Santa Pola saltern (Spain) with 33% salinity (SRX347883) [3]; SS37: metagenome from Santa Pola saltern (Spain) with 37% salinity (SRX090229) [1]; LM18: metagenome from Lake Meyghan (Iran) with 18% salinity (ERS1455390) [4]; LM30: metagenome from Lake Meyghan (Iran) with 30% salinity (ERS1455391) [4]; IC21: metagenome from Isla Cristina saltern (Spain) with 21% salinity [5,6]; SMO1: metagenome from Odiel saltmarshes hypersaline soil, 24 mS/cm conductivity (SRR5753725) [7]; SMO2: metagenome from Odiel saltmarshes hypersaline soil, 54 mS/cm conductivity (SRR5753724) [7].

Supplementary references

1. Fernández, A.B. Ghai, R., Martin-Cuadrado, A.-B., Sánchez-Porro, C., Rodriguez-Valera, F. and Ventosa, A. (2014a). Prokaryotic taxonomic and metabolic diversity of an intermediate salinity hypersaline habitat assessed by metagenomics. *FEMS Microbiol. Ecol*. 88, 623-635.
2. Ghai, R., Pašić, L., Fernández, A.B., Martin-Cuadrado, A.-B., Mizuno, C.M., McMahon, K.D., Papke, R.T., Stepanauskas, R., Rodriguez-Brito, B., Rohwer, F., Sánchez-Porro, A., Ventosa, A. and Rodríguez-Valera, F. (2011). New abundant microbial groups in aquatic hypersaline environments. *Sci. Rep*. 1, 135.
3. Fernández, A.B., Ghai, R., Martin-Cuadrado, A.-B., Sánchez-Porro, C., Rodriguez-Valera, F., and Ventosa A. (2013). Metagenome sequencing of prokaryotic microbiota from two hypersaline ponds of a marine saltern in Santa Pola, Spain. *Genome Announc*. 1, e00933-13.
4. Naghoni, A., Emtiazi, G., Amoozegar, M.A., Cretoiu, M.S., Stal, L.J., Etemadifar, Z, Shahzadeh Fazeli, S.A. and Bolhuis, H. (2017). Microbial diversity in the hypersaline Lake Meyghan, Iran. *Sci. Rep*. 7, 11522.
5. Fernández, A.B., León, M.J., Vera, B., Sánchez-Porro, C. and Ventosa, A. (2014b). Metagenomic sequence of prokaryotic microbiota from an intermediate-salinity pond of a saltern in Isla Cristina, Spain. *Genome Announc*. 2, e00045-14.
6. Fernández, A.B., Vera-Gargallo, B., Sánchez-Porro, C., Ghai, R., Papke, R.T., Rodriguez-Valera, F. and Ventosa A. (2014c). Comparison of prokaryotic community structure from Mediterranean and Atlantic saltern concentrator ponds by a metagenomic approach. *Front. Microbiol*. 5, 196.
7. Vera-Gargallo, B. and Ventosa, A. (2018). Metagenomic insights into the phylogenetic and metabolic diversity of the prokaryotic community dwelling in hypersaline soils from the Odiel Saltmarshes (SW Spain). *Genes* 9, 152.
